# Lower-cholesterol formula feeding alters the early-life cholesterol-bile acid axis in neonatal piglets

**DOI:** 10.1038/s41538-026-01019-8

**Published:** 2026-07-22

**Authors:** Xuan He, Carolyn M. Slupsky

**Affiliations:** 1 Department of Nutrition, University of California, Davis, Davis, CA USA; 2 Department of Food Science and Technology, University of California, Davis, Davis, CA USA

**Keywords:** Biochemistry, Gastroenterology, Molecular biology, Physiology

## Abstract

Infancy is a period of rapid growth with high cholesterol demand, yet vegetable oil-based infant formulas provide substantially less cholesterol than mammalian milk. To characterize metabolic adaptations associated with lower cholesterol formula feeding, we performed a pathway-focused transcriptomic analysis, supported by targeted metabolomics, in neonatal piglets comparing formula-fed and sow-fed groups. Formula feeding consistently induced upregulation of *SREBF2*-associated cholesterol biosynthetic genes in liver and ileum, along with altered expression of genes involved in cholesterol transport and lipoprotein assembly. Bile acid biosynthesis and transport pathways were also upregulated, reflecting a compensatory response favoring bile acid production and conservation. Despite this, luminal bile acid concentrations were reduced, accompanied by lower intestinal *FGF19* signaling and reduced jejunal expression of genes involved in fatty-acid uptake and lipid processing. Together, these findings indicate that lower-cholesterol formula feeding triggers an early-life adaptive response along the cholesterol-bile acid axis, suggesting that dietary cholesterol warrants attention as a component of infant formula.

## Introduction

Cholesterol has traditionally been discussed through the lens of adult cardiovascular disease. Although elevated circulating total cholesterol is associated with cardiovascular disease risk^1^, accumulating evidence indicates that dietary cholesterol intake alone is not a primary driver of atherosclerosis risk^2^; rather, risk is more closely linked to the regulation of cholesterol metabolism, transport, and homeostasis^3^. In parallel, the Developmental Origins of Health and Disease (DoHaD) paradigm highlights how nutritional perturbation during early life, including infancy, may shape long-term metabolic regulation^4,5^. This evolving understanding has brought attention back to early dietary cholesterol exposure, a key component of mammalian milk.

Cholesterol is an essential lipid for mammalian life, especially during infancy, serving as an indispensable building block for the cell membrane, a precursor for the biosynthesis of bile acids and steroid hormones, a component of myelin in the central nervous system, and a critical part of metabolism. For breastfed infants, human milk is a rich dietary source of cholesterol, with a reported average concentration of approximately 16 mg/dL (95% CI 12–20 mg/dL)^6^. Its cholesterol content varies with lactation stage, geographic region and maternal factors, such as ethnicity, circulating cholesterol, and diet^6–8^. In contrast, typical cow’s milk protein-based infant formulas formulated with vegetable oils contain substantially lower amounts of cholesterol (<3 mg/dL), primarily originating from residual milk fat present in the protein source^9^.

Approximately 80% of cholesterol in human milk is associated with the milk fat globule membrane (MFGM), a bioactive structure increasingly recognized for its functional importance in early-life nutrition but historically absent from standard infant formulas^10^. Consistent with these differences in dietary cholesterol level, formula-fed infants exhibit significantly lower circulating total cholesterol concentrations compared with breastfed infants. In contrast, enrichment of vegetable oil-based formulas with cholesterol or cholesterol-containing bovine MFGM leads to circulating cholesterol levels that more closely resemble those observed in breastfed infants^11–14^.

In healthy term infants, high dietary cholesterol intake suppresses endogenous cholesterol synthesis through feedback regulation. Formula-fed infants with low exogenous cholesterol exposure exhibit higher fractional cholesterol synthesis rates than breastfed infants, reflecting a compensatory endogenous cholesterol production^12,14,15^. It has been postulated that elevated cholesterol biosynthesis during infancy may be linked to higher circulating cholesterol concentrations later in life^16–18^. However, the molecular basis of nutritional programming related to early-life cholesterol metabolism remains poorly characterized.

The neonatal piglet provides a unique and physiologically relevant model to investigate these mechanisms in ways not feasible in human studies. Neonatal piglets closely resemble human infants in gastrointestinal development, digestion, and cholesterol metabolism^19,20^, while also allowing controlled formula feeding that closely mimics infant feeding practices. Importantly, cholesterol concentrations in sow milk (approximately 13 mg/dL) are comparable to those in human milk^21,22^, and piglets fed low-cholesterol, vegetable oil-based formulas exhibit lower circulating cholesterol levels than sow-fed counterparts^21–23^, mirroring the observations in human infants. These characteristics provide a strong rationale for using the neonatal piglet to examine cholesterol-related metabolic pathways in early life.

Here, we conducted a pathway-focused secondary analysis by leveraging transcriptomic and metabolomic data from a neonatal piglet feeding study employing scheduled manual bottle-feeding that closely reflects human formula feeding patterns^24,25^. In this study, “low-cholesterol formula” describes the lower cholesterol content of the vegetable-oil-based formulas relative to sow milk, not a comparison among formulas that differed only by cholesterol content. By comparing sow-fed and formula-fed piglets, we aimed to elucidate how reduced dietary cholesterol exposure during infancy reshapes cholesterol and bile acid homeostasis pathways. These findings provide molecular-level mechanistic insight into systemic adaptations associated with formula feeding, while raising important questions about the adequacy of cholesterol provision during early life.

## Results

Detailed differential-expression results for curated genes relevant to cholesterol biosynthesis, cholesterol transport and lipoprotein assembly, bile acid metabolism, and related lipid metabolic pathways are summarized in Suppl. Tables 1–4. Because intestinal cholesterol and bile acid pathways may be regionally specialized, diet effects were evaluated using both pooled small-intestinal models and site-specific models for jejunum and ileum.

### Formula-feeding induces SREBP2-mediated cholesterol biosynthesis

**Fig. 1:**
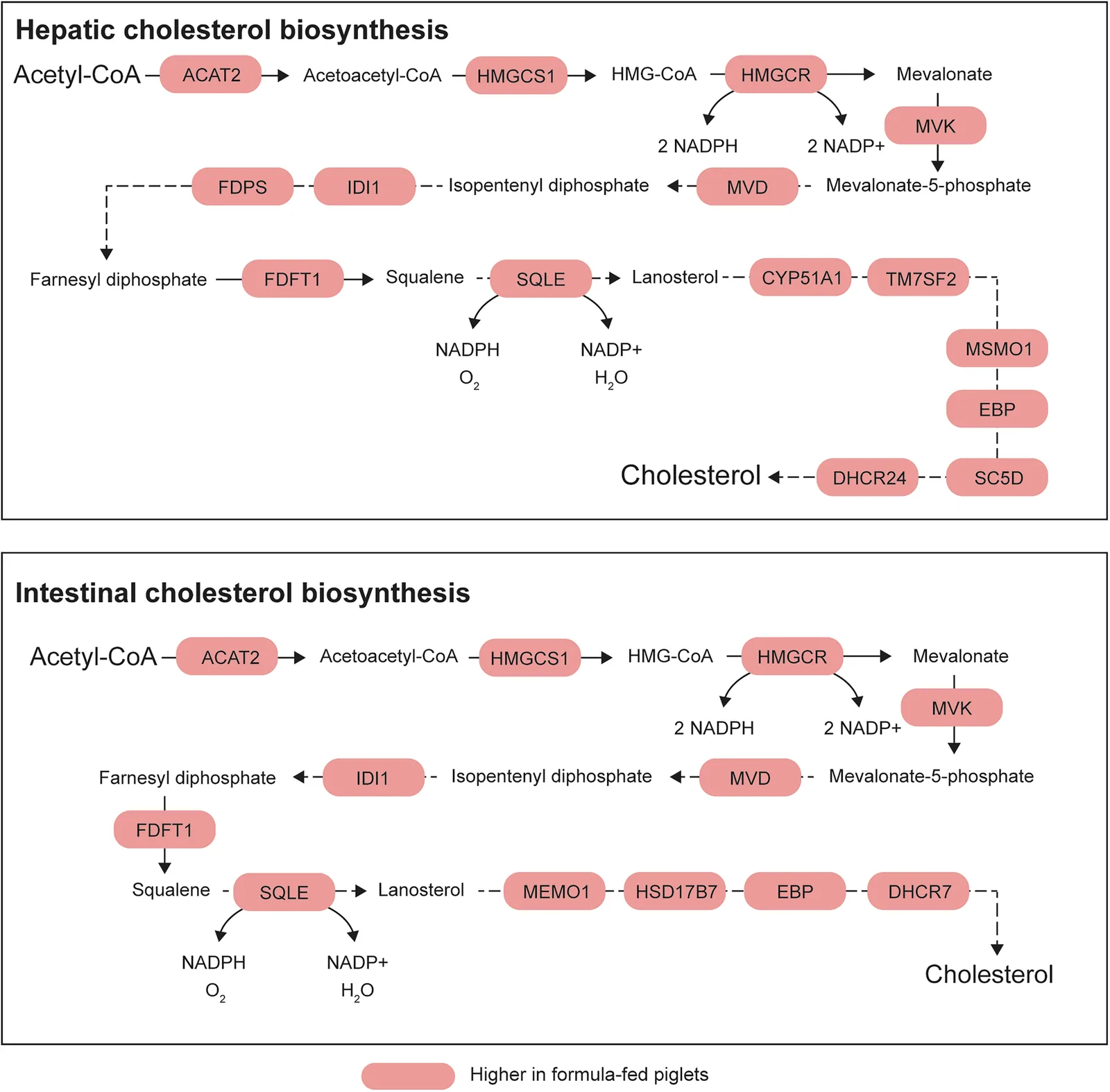
Differential expression of cholesterol biosynthesis genes in liver and small intestine. Pathway schematic of cholesterol biosynthesis highlighting genes identified as differentially expressed in RNA-seq analysis comparing formula-fed and sow-fed piglets. Red indicates higher expression in formula-fed piglets; no genes were decreased relative to sow-fed controls. Detailed differential expression statistics are provided in Supplementary Table 1.

Although all nucleated mammalian cells can synthesize cholesterol, both liver and intestine are the major contributors to endogenous cholesterol production. Here, through RNA-seq-based transcriptome analysis of the liver and small intestine, we found a significantly higher expression of cholesterol biosynthesis genes in formula-fed piglets compared to their sow-fed counterparts (Fig. 1). In parallel, *SREBF2* (sterol regulatory element binding protein type 2; SREBP2), a master transcriptional regulator of cholesterol biosynthesis, was increased in liver. No overall difference in *SREBF2* expression was detected in the pooled small intestine; however, ileum expression was significantly higher in formula-fed piglets, suggesting that the intestinal SREBP2-related response may be regionally concentrated in the ileum. In liver and ileum, *INSIG1* (insulin induced gene 1; INSIG-1) was also upregulated, consistent with a feedback regulatory response associated with SREBP activation^26^. Together, these findings suggest activation of SREBP2-mediated cholesterol biosynthesis as a compensatory response to the low cholesterol content of formula.

**Fig. 2:**
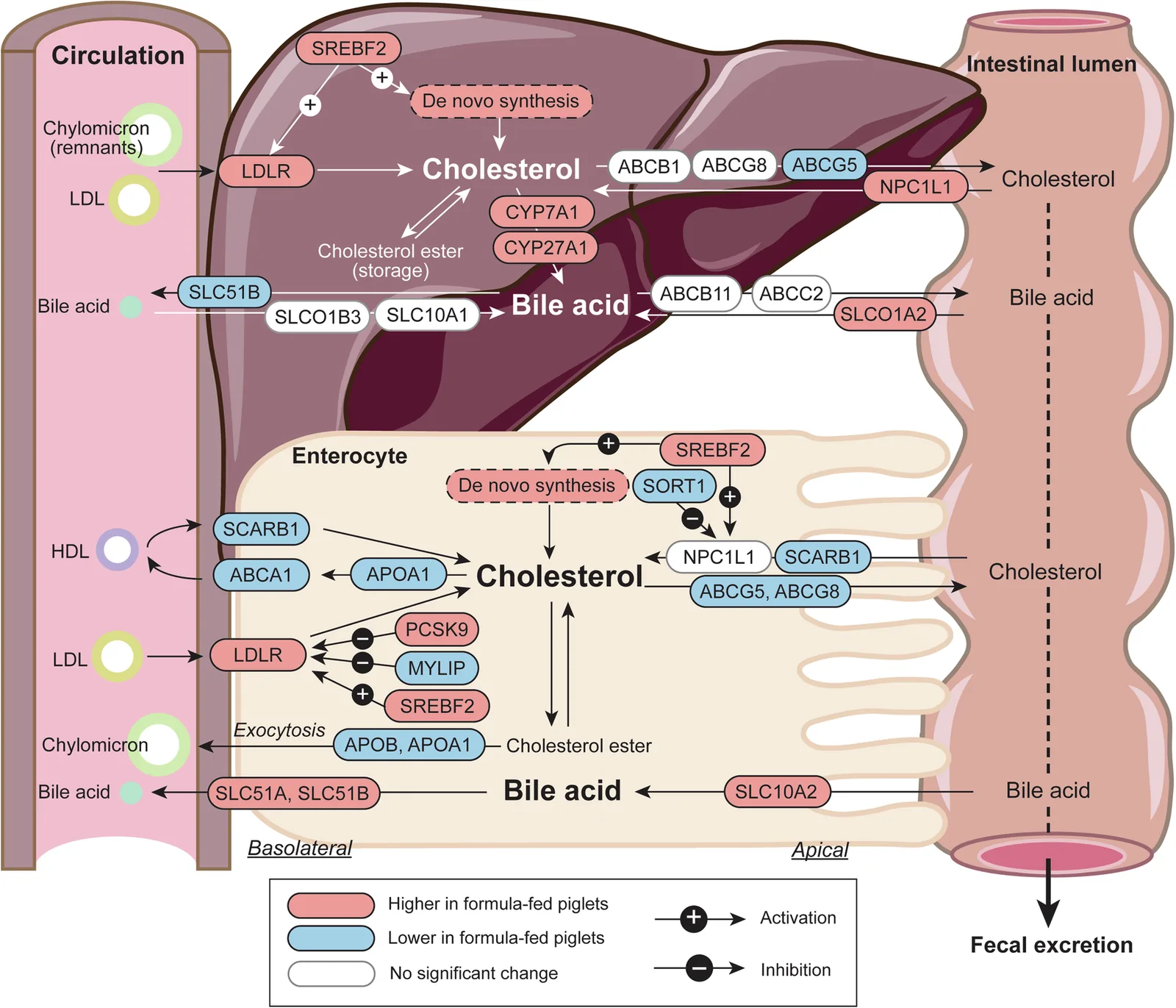
Transcriptional changes across the cholesterol–bile acid axis. Schematic representation of cholesterol metabolism and bile acid synthesis in the liver, biliary secretion into the intestinal lumen, and enterohepatic transport between enterocytes and the portal circulation. Gene expression differences from RNA-seq analysis comparing formula-fed and sow-fed piglets are indicated by color coding (red, higher in formula-fed; blue, lower in formula-fed; uncolored, no significant change). Detailed differential expression statistics are provided in Suppl. Tables 1–3.

Because accumulation of free (unesterified) cholesterol can disrupt cellular membranes, mammalian tissues tightly regulate and integrate cholesterol synthesis with transport, uptake, and efflux. Given the increased expression of cholesterol biosynthesis genes observed in formula-fed piglets, we next examined whether formula-feeding altered cholesterol homeostasis pathways in the small intestine and liver. An integrated overview of transcriptional changes across the cholesterol-bile acid axis presented in this study is illustrated in Fig. 2.

### Formula feeding alters regulation of cholesterol homeostasis in the small intestine

To determine whether formula feeding altered intestinal cholesterol uptake, efflux, or both, we examined the expression of key cholesterol transporters in the small intestine. Expression of *NPC1L1* (Niemann-Pick C1-Like 1), the primary apical transporter mediating intestinal cholesterol uptake, did not differ between formula-fed and sow-fed piglets. Although the *NPC1L1* transcript levels were unchanged, its expression is known to be sensitive to luminal nutrient availability, as well as meal timing and volume^27^, factors that were experimentally controlled only in formula-fed piglets and not in sow-fed piglets. Notably, among regulatory genes linked to *NPC1L1* expression, *SREBF2* expression was higher in the ileum of formula-fed piglets, whereas *SORT1* (sortilin 1) expression was lower across the pooled small intestine, with consistent decreases observed in both the jejunum and ileum. In addition, expression of *SCARB1* (scavenger receptor class B member 1; SR-B1) was significantly lower in the small intestine of formula-fed piglets, with site-specific analyses confirming this reduction in both the jejunum and ileum.

Concurrently, expression of the apical sterol efflux transporters *ABCG5* and *ABCG8* (ATP-binding cassette subfamily G member 5 and 8) was significantly lower in formula-fed piglets in the pooled small-intestine analysis. Site-specific analyses confirmed reduced *ABCG5* expression in both jejunum and ileum, whereas reduced *ABCG8* expression was detected only in the jejunum. Together, these changes in cholesterol uptake and efflux regulation led us to hypothesize that intracellular cholesterol handling may also be altered by formula feeding.

Enterocytes convert free cholesterol derived from intestinal uptake or de novo synthesis into cholesteryl esters, a process that helps prevent accumulation of unesterified cholesterol and supports chylomicron assembly for lymphatic transport. We therefore assessed expression of genes involved in cholesterol esterification and chylomicron assembly. *SOAT1* (sterol O-acyltransferase 1), which catalyzes cholesterol esterification, did not differ in the pooled small-intestine analysis but was significantly higher in the ileum of formula-fed piglets, whereas *SOAT2* (sterol O-acyltransferase 2) did not differ significantly in the small intestine. In parallel, several genes related to chylomicron assembly and lipid transport, including *APOA4* (ApoA-IV) and *APOB* (ApoB-48) were significantly lower in formula-fed piglets in the pooled small-intestine analysis. Together, these findings suggest that formula feeding alters transcriptional regulation of cholesterol esterification and chylomicron-associated lipid transport pathways.

In addition to chylomicron assembly, enterocytes can also direct cholesterol toward nascent high-density lipoprotein (HDL) particles. To assess this pathway, we examined genes involved in HDL-associated cholesterol efflux. Expression of *ABCA1* (ATP-binding cassette subfamily A member 1) and *APOA1*, which support enterocyte cholesterol efflux and nascent HDL formation, was lower in formula-fed piglets, with similar patterns in both jejunum and ileum. Concurrently, *SCARB1* expression was also lower in formula-fed piglets; however, because *SCARB1* may participate in both basolateral HDL interactions and apical sterol flux, its specific functional contribution cannot be inferred from transcriptomic data alone. Collectively, these findings suggest altered transcriptional regulation of HDL-associated cholesterol trafficking in small intestinal enterocytes in formula-fed piglets.

We next examined the LDLR-PCSK9 axis to assess whether LDL receptor-mediated lipoprotein handling was altered in the small intestine. Expression of *LDLR* (low-density lipoprotein receptor), which mediates basolateral uptake of circulating low-density lipoprotein (LDL) cholesterol into enterocytes, was higher in the small intestine of formula-fed piglets, with consistent differences observed in both jejunum and ileum. This pattern was accompanied by changes in multiple regulatory genes. Specifically, *SREBF2*, a known transcriptional activator of *LDLR*^28^, was increased in the ileum of formula-fed piglets, whereas *PCSK9* (proprotein convertase subtilisin/kexin type 9), which promotes lysosomal degradation of LDLR^29^, was also higher in the pooled small-intestine analysis, largely driven by the ileum. In contrast, *MYLIP* (myosin regulatory light chain interacting protein; IDOL), an E3 ubiquitin ligase that targets LDLR for degradation, was lower in formula-fed piglets in the pooled small-intestine analysis. Together, these findings suggest altered transcriptional regulation of LDLR-associated cholesterol uptake pathways in the small intestine with formula feeding.

### Formula feeding alters regulation of cholesterol homeostasis in the liver

To assess whether hepatic uptake of circulating lipoprotein-derived cholesterol was altered with formula feeding, we examined genes involved in LDLR-mediated lipoprotein uptake. Hepatic *LDLR*, a transcriptional target of SREBP2^28^ that mediates uptake of LDL particles and apoE-rich chylomicron remnants, was significantly higher in formula-fed piglets than in sow-fed controls, consistent with the observed increase in hepatic *SREBF2* expression. In contrast, *PCSK9*, which promotes LDLR degradation^29^, did not differ between groups. Together, these findings may reflect increased transcriptional capacity for hepatic receptor-mediated uptake of circulating lipoproteins to compensate for low dietary cholesterol.

In addition to LDLR-mediated lipoprotein uptake, the liver regulates cholesterol homeostasis through biliary export and possible reuptake. Expression of the biliary sterol transporter *ABCG5* was lower in the formula-fed piglets, whereas transporters *ABCG8* and *ABCB1* did not differ between formula-fed and sow-fed piglets. In contrast, hepatic *NPC1L1*, which mediates hepatic cholesterol uptake and has been reported to be transcriptionally regulated by SREBP2^30,31^, was significantly elevated in formula-fed piglets. Collectively, these transcriptional changes may suggest a reduced biliary cholesterol export and increased biliary cholesterol reuptake in formula-fed piglets.

### Hepatic bile acid biosynthesis and FXR-FGF19 signaling

In the liver, cholesterol is converted to bile acids through the classical and alternative pathways, regulated by the rate-limiting enzymes cholesterol 7α-hydroxylase (CYP7A1) and sterol 27-hydroxylase (CYP27A1), respectively. Following synthesis, bile acids are secreted into bile via the bile salt export pump (BSEP; gene symbol *ABCB11*) as well as through transporters in the multidrug resistance-associated protein (MRP; *ABCC*) and organic anion transporting polypeptide (OATP; *SLCO*) families, and are subsequently delivered to the intestinal lumen, where they act as surfactants to facilitate lipid digestion and absorption through micellar solubilization. Because bile acids are synthesized directly from cholesterol, we hypothesized that bile acid metabolism and its regulatory pathways may also be altered in formula-fed piglets.

In the liver, expression of *CYP7A1* and *CYP27A1* was elevated in formula-fed piglets. In contrast, the hepatic expression of *ABCB11* and *ABCC2* (ATP binding cassette subfamily C member 2; MRP2) remained unchanged. Expression of *SLCO1A2* (solute carrier organic anion transporter family member 1A2; OATP1A2), which mediates bile acid reabsorption from bile to cholangiocytes, was higher in formula-fed piglets. Meanwhile, hepatic expression of *SLC51B* (solute carrier family 51 subunit beta, OST-β), which facilitates bile acid efflux from hepatocytes into the blood, was lower in the formula-fed piglets. Hepatic expression of *SLCO1B3* (organic anion transporting polypeptide 1B3; OATP1B3) and *SLC10A1* (sodium taurocholate cotransporting polypeptide; NTCP), which mediate bile acid uptake from portal blood into hepatocytes, was not significantly altered.

**Fig. 3:**
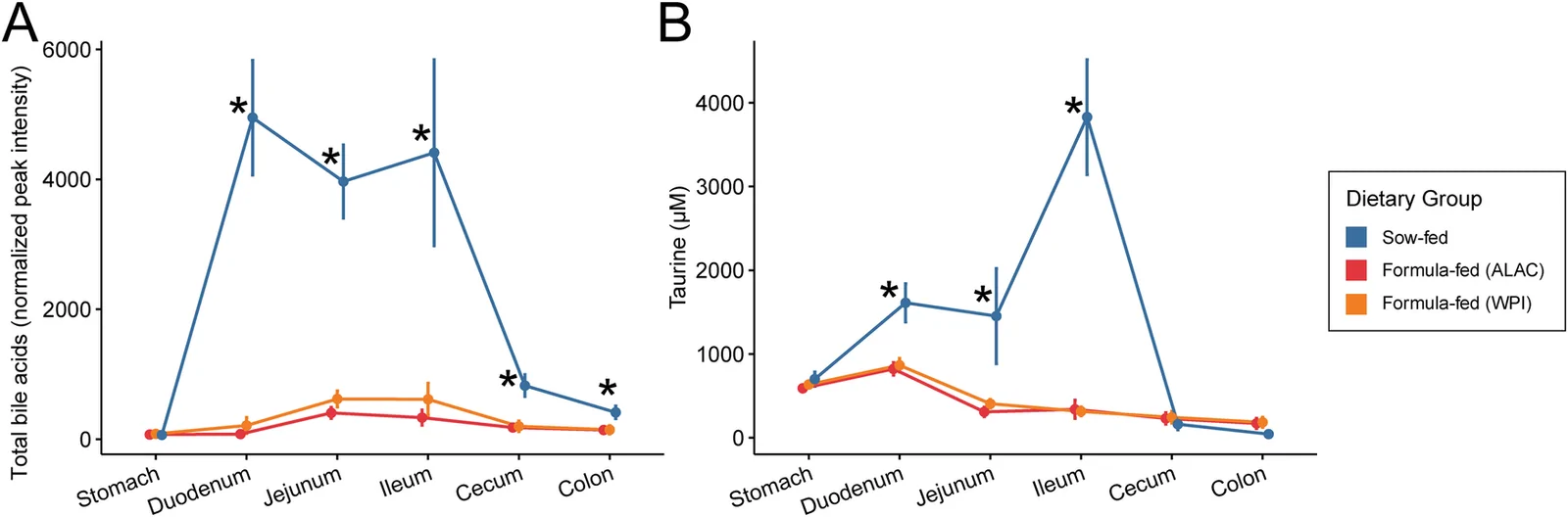
Distribution of total bile acids and taurine along the gastrointestinal tract. **A** Total bile acids and (**B**) taurine concentrations across gastrointestinal segments in sow-fed, α-lactalbumin-enriched formula-fed (ALAC), and whey protein isolate-based formula-fed (WPI) piglets. Data are displayed by original dietary group assignment for transparency. Asterisks (*) indicate significant differences between the sow-fed piglets and pooled formula-fed piglets, defined as the equal-weighted mean of ALAC and WPI compared with the sow-fed piglets within each gastrointestinal segment ([*(0.5 × ALAC* *+* *0.5 × WPI) versus sow-fed*], FDR-adjusted *p*-value < 0.05). The ALAC vs WPI contrasts were evaluated, with no significant differences observed within any gastrointestinal segment. Line plots are shown as mean ± SEM.

Consistent with these transcriptional changes, total bile acid levels in the small intestinal lumen were markedly lower in formula-fed piglets than in sow-fed controls, as quantified by ^1^H NMR metabolomics analysis (Fig. 3A). In the small intestine, expression of the bile acid transporters *SLC10A2* (solute carrier family 10 member 2; ASBT), *SLC51A* (solute carrier family 51 subunit alpha; OST-α), and *SLC51B* was higher in formula-fed piglets in the pooled small-intestine analysis. These transporters facilitate bile acid reabsorption from the intestinal lumen into the portal circulation. Site-specific analyses showed that increases in *SLC51A* and *SLC51B* occurred in both the jejunum and ileum, whereas the increase in *SLC10A2* expression was primarily driven by the ileum. Additionally, similar reductions in serum, biliary, and fecal bile acid levels have been reported in other piglet studies of low-cholesterol formula feeding^21,32^. Collectively, these changes reflect an adaptive response linking cholesterol availability and synthesis to enhanced conservation of bile acids through increased intestinal recycling.

In addition to reduced luminal bile acid levels, taurine in the intestinal digesta was significantly lower in formula-fed piglets, with the largest difference observed in the ileum (Fig. 3B). In neonatal piglets, luminal bile acid species occur in both glycine- and taurine-conjugated forms; however, luminal glycine can be strongly influenced by protein digestion. Therefore, changes in luminal taurine may provide a more specific readout of altered bile acid turnover and microbial deconjugation in the distal small intestine, offering additional support for altered luminal bile acid processing in formula-fed piglets.

Reduced luminal bile acid availability is known to attenuate activation of the farnesoid X receptor (FXR) in enterocytes, leading to decreased expression of its downstream target fibroblast growth factor 19 (FGF19). In the present study, intestinal *FGF19* expression was significantly reduced in formula-fed piglets, indicating a reduction in ligand-dependent FXR signaling. Notably, FXR mRNA expression (gene symbol *NR1H4*) was increased, reflecting an adaptive shift toward ensuring FXR sensitivity under reduced bile acid availability. In the liver, expression of *FGF21* (fibroblast growth factor 21) in the formula-fed piglets was lower, but did not meet the predefined significance or fold-change cut-off (adjusted *p*-value = 0.053; shrunken log2 fold change = −0.53).

**Fig. 4:**
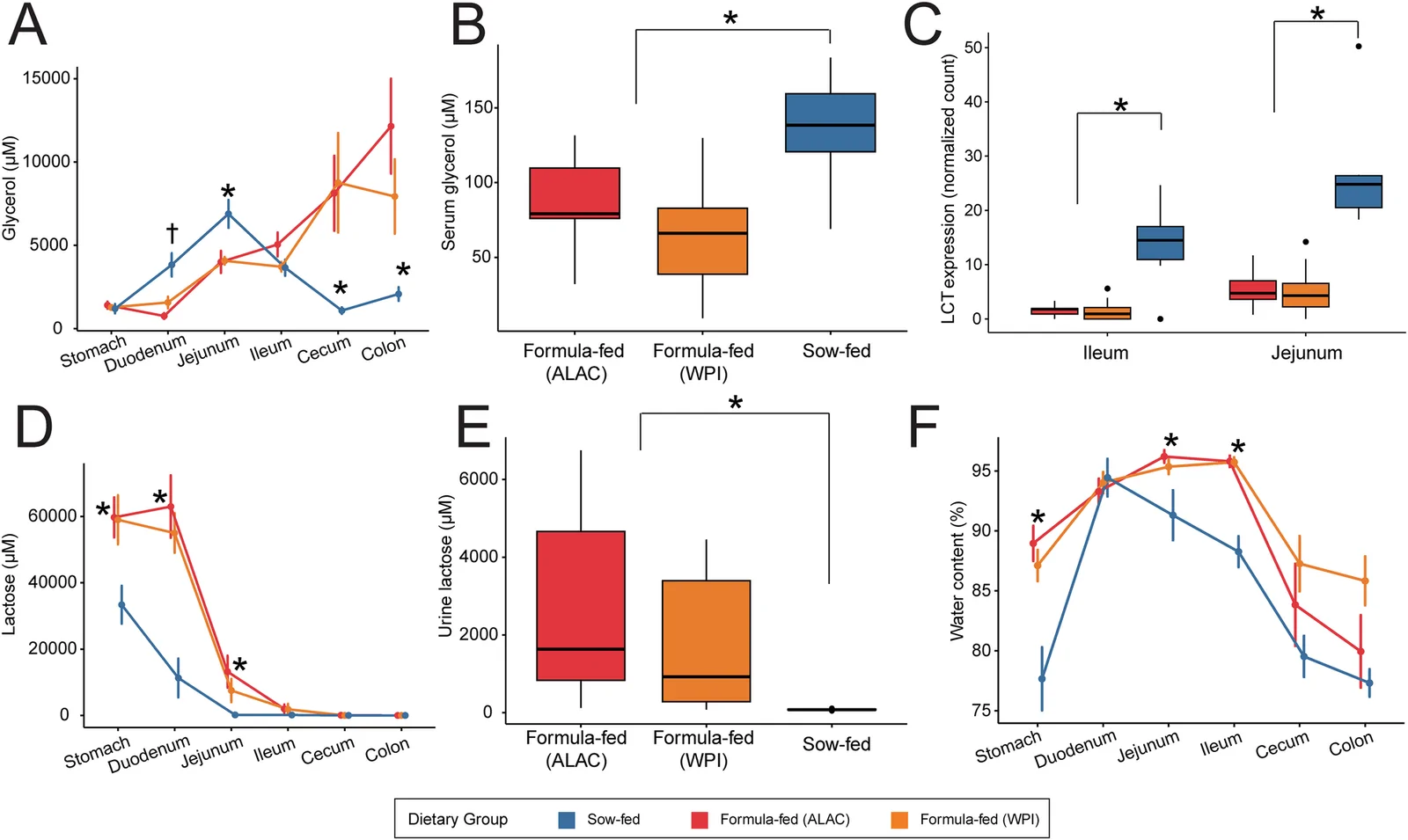
Effects of formula feeding on glycerol, lactose, lactase expression, and intestinal water. **A** Glycerol concentrations across gastrointestinal segments and (**B**) serum glycerol concentrations in sow-fed, α-lactalbumin-enriched formula-fed (ALAC), and whey protein isolate-based formula-fed (WPI) piglets. **C** *LCT* (lactase) expression in jejunum and ileum (DESeq2 normalized counts). **D** Gastrointestinal lactose concentrations and (**E**) urinary lactose concentrations. **F** Gastrointestinal water content. Data are displayed by original dietary group assignment for transparency. Asterisks (*) and daggers (†) indicate significant (FDR-adjusted *p*-value < 0.05) and marginally significant (FDR-adjusted *p*-value < 0.10) differences, respectively, between sow-fed piglets and pooled formula-fed piglets, defined as the equally weighted mean of ALAC and WPI. Among comparisons showing a significant pooled formula-fed vs sow-fed difference, no significant ALAC vs WPI differences were detected. Line plots represent mean ± SEM.

Given the central role of bile acids in micellar solubilization, intestinal lipid digestion, and absorption, reduced luminal bile acid availability in formula-fed piglets may be associated with altered lipid utilization. Fatty acid transport protein 4 (FATP4), located on the apical brush border of intestinal epithelial cells, plays a key role in enterocyte uptake of long-chain fatty acids. Intestinal expression of *FATP4* was significantly lower in formula-fed piglets, with the difference driven largely by the jejunum. In addition, jejunal expression of *FABP2* (fatty acid binding protein 2), *DGAT1* (diacylglycerol O-acyltransferase 1), *CIDEB* (cell death inducing DFFA like effector B) and *MTTP* (microsomal triglyceride transfer protein) was lower in formula-fed piglets than in sow-fed counterparts. These genes function in intracellular fatty-acid trafficking, triacylglycerol re-esterification, and chylomicron assembly. Jejunal PRKAA2 (protein kinase AMP-activated catalytic subunit alpha 2; AMPKα2) transcript abundance was also lower in formula-fed piglets, consistent in direction with altered long-chain fatty-acid uptake. Glycerol, a product of triglyceride hydrolysis, was lower in the duodenal and jejunal contents and in serum of formula-fed piglets, but higher in the cecum and colon, suggesting greater release of glycerol from unabsorbed triglycerides in the distal gut (Fig. 4A, B). Taken together, these findings point to altered intestinal lipid absorption and processing in formula-fed piglets, potentially related to reduced bile acid availability.

### Intestinal HNF1A downregulation and lactase-associated responses

Hepatocyte nuclear factor-1α (HNF-1α; gene symbol *HNF1A*) is an intestinal transcription factor that regulates cholesterol and bile acid metabolism and homeostasis. In the small intestine of formula-fed piglets, *HNF1A* expression was lower, with the difference primarily driven by the ileum. This intestine-specific shift occurred alongside increased ileal expression of *SREBF2* and increased expression of *SLC10A2* (ASBT), consistent with known regulatory role of HNF-1α in controlling SREBF2^33^ and SLC10A2^34^ expression.

Furthermore, HNF-1α has also been shown to regulate lactose digestion through control of lactase (LPH; gene symbol *LCT*)^35,36^. Consistent with the reduction in *HNF1A* expression, *LCT* expression was lower in formula-fed piglets, with differences observed in both the jejunum and ileum (Fig. 4C). This was accompanied by higher lactose abundance in small intestinal contents and urine (Fig. 4D, E), consistent with reduced lactose hydrolysis. Finally, because HNF-1α also influences intestinal epithelial cell growth and regulates water absorption in the intestine^37^, we measured percent water content in luminal digesta across the gastrointestinal tract. Consistent with this, digesta water content was modestly higher in formula-fed piglets than in sow-fed controls (Fig. 4F), suggesting a potential shift in HNF-1α-mediated intestinal adaptation related to water absorption.

## Discussion

Early infancy is a critical period of rapid growth and tissue expansion, during which extensive cellular proliferation, differentiation, and membrane biogenesis create substantial demand for cholesterol. In addition, cholesterol serves as a precursor for bile acids, steroid hormones, and vitamin D, all of which are essential during early development. In mammalian milk, cholesterol is naturally delivered as part of the MFGM^10^; however, typical infant formulas are manufactured using shelf-stable vegetable oils and therefore contain substantially lower levels of cholesterol^9^. At the same time, nutritional understanding of cholesterol has evolved, particularly in adults, in whom circulating cholesterol is determined predominantly by endogenous synthesis and cholesterol homeostasis rather than directly by dietary cholesterol intake^38^. In contrast, during infancy, a period of elevated anabolic demand, the relative contributions of dietary and endogenous cholesterol are not well established. Formula-fed infants exhibit lower circulating total cholesterol concentrations and higher fractional cholesterol synthesis than breastfed infants^12,14,15^, suggesting a compensatory shift in the balance between cholesterol supply and synthesis under conditions of low dietary cholesterol exposure. Here, we address this gap by performing a pathway-focused transcriptomic analysis, supported by metabolomic measurements, to characterize changes in cholesterol and bile acid metabolism and homeostasis in an early-life formula-feeding model. Because cholesterol and bile acid-related changes were directionally consistent across the two formula-fed groups included in the original study design, with no evidence that protein source explained the main effects, we focus our interpretation on the shared formula-feeding exposure.

In the present study, we observed a consistent upregulation of SREBP2-mediated cholesterol biosynthesis in both the liver and ileum of formula-fed piglets. This transcriptional activation aligns with previous neonatal piglet studies using vegetable oil-based, low-cholesterol formulas^21–23,32,39^ and mirrors observations from stable isotope studies in human infants^12,14,15^. Collectively, these findings support an adaptive shift toward enhanced endogenous cholesterol biosynthesis in response to limited dietary cholesterol from formula feeding.

This shift toward increased cholesterol biosynthesis was coupled with transcriptional changes in intestinal sterol transporter expression. Specifically, formula-fed piglets exhibited reduced enterocytic expression of *ABCG5* and *ABCG8*, suggesting attenuation of cholesterol loss under low dietary cholesterol conditions. Although a previous neonatal piglet study reported increased ileal NPC1L1 expression under formula feeding^32^, NPC1L1 transcript abundance did not differ in our study. However, increased intestinal *SREBF2* (a transcriptional activator of NPC1L1^40^), alongside reduced *SORT1* (a reported suppressor^41^) suggests a regulatory context permissive to enhanced cholesterol uptake without requiring overt upregulation of NPC1L1 transcription. In parallel, reduced expression of enterocytic *SCARB1* (SR-B1) was observed in formula-fed piglets. While the role of SR-B1 in intestinal cholesterol absorption remains debated^42^, both SR-B1 and NPC1L1 facilitate uptake of non-cholesterol sterols, including phytosterols, as well as fat-soluble vitamins^42^, suggesting broader effects on intestinal sterol handling beyond cholesterol alone. Because phytosterols and fat-soluble vitamins were not evaluated in the present study, it remains unclear whether they contributed to the observed transporter-related adaptations or were themselves affected by altered intestinal transporter expression.

In the context of enterocytic lipoprotein assembly and export, expression of genes encoding key apolipoproteins and transporters involved in lipoprotein formation and secretion, including *APOB*, *APOA1*, and *ABCA1*, was reduced in the small intestine of formula-fed piglets. These transcriptional changes suggest modulation of enterocytic lipoprotein assembly and export, potentially favoring intracellular cholesterol retention over secretion into circulating lipoproteins under conditions of limited dietary cholesterol availability.

In the liver, formula feeding led to increased expression of *LDLR*, consistent with findings from another neonatal piglet study using commercial infant formulas^32^. Hepatic expression of *NPC1L1*, a transporter involved in cholesterol reuptake from the biliary compartment, was also elevated. Because both *LDLR* and *NPC1L1* are transcriptionally regulated by SREBP2^28,30,31^, their upregulation likely reflects activation of a shared regulatory mechanism mediating cholesterol biosynthesis. Functionally, increased hepatic *NPC1L1* expression may serve to reduce biliary cholesterol excretion by promoting reabsorption, which is in agreement with previously published studies demonstrating reduced biliary cholesterol excretion in formula-fed piglets^21,23^. The lower hepatic expression of *ABCG5* observed in formula-fed piglets should be interpreted cautiously, because ABCG5 and ABCG8 function as a heterodimer and hepatic expression of *ABCG8* was not significantly altered. This discordance in hepatic cholesterol efflux transporter expression warrants further validation beyond transcript-level assessment. Nevertheless, the lower hepatic *ABCG5* expression in formula-fed piglets, together with higher hepatic *NPC1L1* expression, forms a directionally consistent pattern suggestive of altered hepatic cholesterol handling that favors cholesterol conservation when exogenous supply is limited.

In parallel with enhanced cholesterol biosynthesis, formula feeding was associated with increased expression of hepatic *CYP7A1*, the rate-limiting enzyme in the classical bile acid synthesis pathway, and hepatic *CYP27A1*, a key enzyme in the alternative bile acid synthesis pathway. Despite this, total bile acid levels in the small intestinal lumen remained reduced. Increased expression of the intestinal bile acid transporters *SLC10A2*, *SLC51A*, and *SLC51B* suggests enhanced enterohepatic recycling and an adaptive response to preserve bile acids. In addition, reduced luminal bile acid levels were accompanied by decreased intestinal *FGF19* expression, suggesting attenuation of ligand-dependent FXR signaling. Similar alterations in bile acid pool size and FXR signaling have been reported in other neonatal piglets fed cholesterol-free or low-cholesterol formulas^32,39^. Collectively, these findings point to a compensatory response favoring bile acid production and conservation; however, this response appears insufficient to maintain luminal bile acid availability under conditions of low dietary cholesterol. Because FXR-FGF19 signaling serves as a key regulatory bridge between intestinal bile acid sensing and hepatic metabolic control, disruption of this pathway may have broader implications for lipid utilization, metabolic homeostasis, and gut-liver communication during early life^43,44^.

This regulatory tension may be especially important during early postnatal development, particularly in human infants, whose disproportionately large and rapidly expanding brain depends on efficient lipid digestion and lipid-derived substrates, including ketone bodies and fatty acids, to support structural growth and high metabolic activity^45,46^. Because neonatal bile acid secretion is relatively immature, lipid digestion in breastfed infants is supported by bile salt-stimulated lipase (BSSL) present in human milk^47^. In contrast, typical formulas do not provide BSSL; accordingly, formula-fed infants have been reported to exhibit greater stool fat loss^48^ and increased formation of calcium-fatty acid soaps, contributing to firmer stools^49^. In this piglet study, lower bile acid concentrations in intestinal digesta were accompanied by reduced jejunal expression of genes involved in lipid uptake and post-digestive processing, including *FABP2*, *DGAT1*, *CIDEB*, and *MTTP*, as well as lower glycerol concentrations in duodenal and jejunal contents and in serum in formula-fed piglets compared with sow-fed counterparts. Taken together, these findings suggest that reduced luminal bile acid availability under low-cholesterol formula feeding may further constrain intestinal lipid handling during a period of increased lipid demand.

Beyond the cholesterol-bile acid adaptations described above, the formula-feeding-associated decrease in intestinal *HNF1A* expression may also extend to lactose metabolism. HNF-1α is a master regulator linked to cholesterol and bile acid homeostasis and gut barrier integrity^33,34,37^, and also acts upstream of *LCT*^35,36^, which is required for lactose digestion. In the present study, lower intestinal *HNF1A* expression in formula-fed piglets was accompanied by lower *LCT* expression in the jejunum and ileum, greater residual lactose abundance in the upper intestinal lumen, and higher urinary lactose, despite comparable lactose content between the formulas and sow milk. This observation suggests that formula feeding may be linked to altered intestinal lactose utilization. However, other formula-related factors, such as differences in gastric emptying rate or matrix-dependent nutrient absorption kinetics, may also influence the timing and efficiency of lactose digestion and absorption. Because urinary lactose can also reflect intestinal permeability and renal solute handling, the mechanism underlying increased urinary lactose is likely multifactorial. Nevertheless, these findings identify a potential link between early-life feeding and HNF-1α-associated regulation that extends beyond the observed cholesterol-bile acid axis. Furthermore, because HNF-1α is also involved in pancreatic insulin production^50^ and regulates the mechanistic target of rapamycin complex 1 (mTORC1) pathway^51^, this unexamined relationship may generate future hypotheses regarding how early feeding shapes systemic metabolic development.

Epidemiological studies have reported that formula feeding in infancy is associated with higher total cholesterol later in life^16,17^. In one cohort, formula feeding was linked to higher LDL-cholesterol concentrations, lower HDL-cholesterol concentrations, and a higher LDL:HDL ratio^52^. Additional evidence for persistent effects of early feeding mode on the cholesterol-bile acid axis comes from non-human primate studies. Formula-fed baboons showed increased cholesterol 7α-hydroxylase activity during milk feeding that persisted after weaning^53^, and later exhibited higher serum cholesterol concentrations and total bile acid synthesis rates at the juvenile stage^54^. Although causality and the responsible nutritional drivers remain uncertain, evidence framed within the DoHaD concept suggests that key regulatory nodes in cholesterol and bile acid metabolism, including *SREBF2*^55^*, LDLR*^56^*, CYP7A1*^57^, and *FGF19*^58^, are developmentally plastic and may be susceptible to epigenetic modification. Whether low dietary cholesterol exposure in early life contributes to persistent epigenetic remodeling of the candidate genes identified in the present study remains an important question for future investigation.

These findings should be viewed in light of several inherent constraints. First, this pathway-focused secondary analysis was based on an existing feeding cohort not designed to directly evaluate dietary cholesterol supplementation. Therefore, the observed responses should be interpreted as formula-feeding-associated adaptations occurring in the context of lower dietary cholesterol exposure, rather than as effects attributable to reduced dietary cholesterol alone. The smaller sample size of the sow-fed group relative to formula-fed groups may have reduced statistical power, potentially limiting our ability to detect some diet-associated differences. Furthermore, although feeding schedules were coordinated using oxytocin administration to approximate timing between groups, precise control of feeding volume and milk intake in sow-fed piglets was not feasible. This variability, inherent to a physiological suckling model, reflects real-world differences between breastfed and formula-fed infants. In addition, the formula used in this study was soybean oil-based and relatively enriched in polyunsaturated fatty acids, such as linoleic acid, whereas sow milk contains higher proportions of saturated fatty acids^59^. Dietary fatty acid composition has been shown to influence hepatic cholesterol metabolism and bile acid pool production^60,61^, and some of the observed differences may therefore reflect the combined effects of cholesterol content and dietary lipid composition. Vegetable oil-based formulas also inherently contain phytosterols, which have been shown to influence cholesterol absorption and homeostasis^62^, but were not quantified in the present study. Their potential contribution to the observed transcriptional adaptations therefore cannot be excluded. Furthermore, this study represents a pathway-focused secondary analysis of existing transcriptomic and metabolomic datasets, and direct quantification of tissue cholesterol concentrations and comprehensive bile acid profiling were not performed. Nonetheless, other neonatal piglet studies have documented differences in circulating cholesterol and bile acid pools under similar feeding conditions^21–23,32^, supporting the biological plausibility of the regulatory adaptations observed here.

Taken together, our findings in neonatal piglets suggest that formula feeding may promote earlier or more pronounced activation of cholesterol and bile acid regulatory pathways. This activation likely represents an adaptive response to limited dietary cholesterol, consistent with the capacity of healthy term infants to adjust cholesterol synthesis in response to intake. However, endogenous cholesterol biosynthesis is metabolically demanding, requiring substantial inputs of acetyl-CoA, ATP, oxygen, and reducing equivalents such as NAD(P)H, and may also be promoted by insulin-responsive mTORC1 signaling^63^. The long-term implications of sustaining elevated endogenous cholesterol synthesis during early life remain unclear. Whether compensatory increases in cholesterol production impose subtle energetic or signaling burdens, or influence later metabolic trajectories, warrants further investigation.

Importantly, typical infant formulas provide substantially less cholesterol than human milk. MFGM supplementation, which reintroduces cholesterol alongside complex milk-derived lipids, has been shown to partially narrow metabolic differences between breastfed and formula-fed infants^64,65^ and to influence circulating cholesterol and lipoprotein levels in preclinical models^66^. Whether such effects reflect partial restoration of cholesterol homeostasis and its downstream bile acid-FXR signaling pathway remains unclear. Although our study does not establish causality or justify changes in clinical practice, it raises a broader question: should dietary cholesterol be considered an indispensable component of the infant diet?

## Methods

### Animal study design overview and data collection

This study represents a post-hoc analysis of data generated from a previously published piglet feeding trial^24,25^. In the original experiment, piglets were allowed to suckle from their sow from birth to Day 6 and were stratified by body weight, sex and litter before random assignment to one of three groups: α-lactalbumin-enriched formula (ALAC; *n* = 12, 6 males and 6 females), whey protein isolate-based formula (WPI; *n* = 12, 6 males and 6 females), or sow-fed controls (*n* = 6, 3 males and 3 females). The ALAC formula contained α-lactalbumin-enriched whey protein (Lacprodan® ALPHA-50, Arla Foods Ingredients, Denmark), whereas the WPI formula contained standard whey protein isolate enriched in β-lactoglobulin (Lacprodan® DI-9224, Arla Foods Ingredients, Denmark) as the primary whey source.

From Days 6-16, ALAC and WPI piglets were group-housed and bottle-fed formula in multiple daily feedings, whereas SF piglets remained with their birth sow. One sow-fed piglet was euthanized on Day 7 due to accidental injury caused by the sow. One female piglet assigned to the ALAC group was reassigned to the sow-fed group because of repeated attempts to return to her sow and refusal to consume the assigned formula. In total, 11 ALAC piglets (6 males, 5 females), 12 WPI piglets (6 males, 6 females), and 6 SF piglets (3 males, 3 females) completed the study. On Day 16, piglets were euthanized following a standardized feeding protocol designed to achieve a similar postprandial sampling time across groups. Sows nursing the sow-fed piglets received 20 USP units of oxytocin 90 min before piglet euthanasia to stimulate milk letdown approximately 60 min prior to euthanasia. The formula-fed piglets were fasted for approximately 5 h, then allowed to consume up to 120 mL of formula within 15 min, and were euthanized approximately 1 h after feeding. Blood, intestinal contents, and tissue samples were collected for downstream analyses.

Animal handling was approved by the University of California, Davis (UC Davis) Institutional Animal Care and Use Committee (IACUC protocol #22011). Detailed descriptions of the animal study procedures, formula composition, growth outcomes, and sample collection protocols have been reported previously^24,25^. RNA-seq transcriptomic and metabolomics datasets generated from this study have also been published^24,25^. The cholesterol and bile acid pathway analyses presented here were not prespecified outcomes of the original animal trial but were conducted as a secondary analysis of previously generated datasets. To provide context for the present pathway-focused secondary analysis, a concise summary of dietary treatment composition is summarized in Suppl. Table 5.

### Differential expression analysis

Prior to differential expression analysis, principal component analysis (PCA) was performed to assess sample clustering and identify potential outliers (Suppl. Fig. 1). RNA-seq differential expression analysis was performed using DESeq2 (v1.50.2)^67^. Gene-level HTSeq counts were analyzed separately for each tissue using a negative binomial generalized linear model with the design formula *~ SowID + Diet*, where *Diet* included three levels: sow-fed, WPI, and ALAC. Inclusion of *SowID* allowed adjustment for litter effects.

Jejunal and ileal samples showed only modest separation in global gene expression profiles compared with colon. Therefore, complementary analytical approaches were implemented for small-intestine tissues. Diet effects were first evaluated using a pooled model including intestinal site (jejunum vs ileum) together with litter (*SowID*) as covariates. To assess potential regional contributions, additional site-specific analyses were performed for jejunum and ileum separately while adjusting for litter effects only. This approach was applied because genes involved in cholesterol and bile acid transport in the small intestine may exhibit site-dependent expression, whereas pooled modeling can increase statistical power for detecting diet-related effects in genes with higher between-animal variability.

To evaluate the overall effect of formula feeding relative to sow milk, we estimated a pooled contrast representing the equally weighted mean of the two formula groups relative to sow-fed: ‘*Formula vs Sow-fed* = *½(WPI vs Sow-fed)* + *½(ALAC vs Sow-fed)’*. In DESeq2, this was implemented using a contrast vector assigning weights of 0.5 to the ‘*WPI vs Sow-fed’* and ‘*ALAC vs Sow-fed’* coefficients. This approach preserves the three-group structure while estimating a single overall formula-feeding effect. To confirm that observed feeding-related effects were not driven by differences between the two formula groups, a direct ALAC vs WPI contrast was evaluated in parallel.

Effect sizes were reported as log2 fold changes with adaptive shrinkage using *lfcShrink(type* = *“ashr”)*^68^. Statistical significance was assessed using Wald tests with Benjamini-Hochberg false discovery rate correction. No additional correction was applied across the liver, pooled small-intestine, jejunum and ileum models. Genes with an adjusted *p*-value < 0.05 and an absolute log2 fold change > 0.585 (> 1.5-fold change) were considered differentially expressed.

### Targeted pathway analysis

In the present study, pathway analyses focused on genes involved in the cholesterol-bile acid axis, including: (a) cholesterol biosynthesis and sterol transport processes that determine substrate availability for bile acid production; (b) hepatic bile acid synthesis, transport, and regulatory signaling involved in enterohepatic circulation; and (c) lipoprotein assembly, remodeling, and receptor-mediated uptake pathways that influence cholesterol distribution and hepatic delivery. Other downstream cholesterol-derived pathways, such as steroid hormone synthesis or vitamin D metabolism, were outside the mechanistic focus of the present study.

To comprehensively curate gene sets relevant to these processes, candidate genes involved in cholesterol metabolism and bile acid homeostasis were compiled from publicly available pathway databases, including Reactome (release 95, December 2025)^69^ and Molecular Signatures Database (MSigDB v2026.1Hs, January 2026). MSigDB gene sets incorporated curated pathway annotations derived from Reactome^69^, KEGG^70^, WikiPathways^71^, and MSigDB^72^. Pathway annotations included both pig-specific Reactome gene sets as well as curated human pathway gene sets through MSigDB. Because pathway resources use different gene identifier systems, human Ensembl gene identifiers were mapped to pig orthologs using biomaRt^73^, which queries the Ensembl BioMart database^74^ for homolog annotation. The resulting gene sets were merged and deduplicated to generate a unified list of pig Ensembl gene identifiers, which was subsequently used to screen differential expression results across tissues. Human-to-pig ortholog mapping was used to improve pathway coverage for candidate genes related to cholesterol metabolism and bile acid homeostasis. Key genes discussed in the Results and Discussion were manually reviewed for known biological function.

### Statistical analysis

Statistical comparisons among dietary groups within each gastrointestinal region and in serum and urine were performed in R using linear regression models on pseudo-log transformed metabolite concentrations. Estimated marginal means were used to compare pooled formula-fed piglets, defined as the equal-weighted mean of ALAC and WPI piglets, with sow-fed piglets [*(0.5* *×* *ALAC* *+* *0.5* *×* *WPI) versus sow-fed*]. A direct ALAC versus WPI contrast was evaluated in parallel. Intestinal water content was assessed using the same approach but without log transformation. *P*-values were adjusted within each tested gastrointestinal region or compartment using the Benjamini-Hochberg method. Data were visualized using the *ggplot2* and *ggpubr* packages. Statistical significance was defined as FDR-adjusted *p*-value < 0.05.

## Supplementary information

Supplementary information

## Data Availability

No new transcriptomic or metabolomic data were generated for the present secondary analysis. The RNA-seq datasets used here, which were generated in a previously published piglet feeding trial^25^, are available in the Gene Expression Omnibus under accession GSE281726. The metabolomics data used in this analysis are available from the cited publication.
