# Supplementary material for: Lower-cholesterol formula feeding alters the early-life cholesterol-bile acid axis in neonatal piglets
Source: NPJ Sci Food. 2026 Jul 22;10:364. doi: 10.1038/s41538-026-01019-8 (PMC13760042; doi:10.1038/s41538-026-01019-8)
Supplement: Supplementary file 1 [file 41538_2026_1019_MOESM1_ESM.pdf]

Supplementary Information

Lower-cholesterol formula feeding alters the early-life cholesterol-bile acid axis in neonatal piglets

Xuan He[1,2], Carolyn M. Slupsky[1,2]

[1] Department of Nutrition, University of California-Davis, Davis, CA, United States
[2] Department of Food Science and Technology, University of California-Davis, Davis, CA, United States

Table of Contents

Supplementary Figure

- **Supplementary Figure 1.** Principal component analysis (PCA) of RNA-seq gene expression data used to assess sample clustering and identify potential outliers.

Supplementary Tables

- **Supplementary Table 1.** Differential expression of genes involved in cholesterol biosynthesis and regulatory pathways.
- **Supplementary Table 2**. Differential expression of genes involved in cholesterol transport and associated lipoprotein assembly.
- **Supplementary Table 3.** Differential expression of genes involved in bile acid biosynthesis, regulation, transport, and detoxification.
- **Supplementary Table 4.** Differential expression of genes involved in other lipid metabolic pathways, including fatty acid uptake, intracellular trafficking, triacylglycerol metabolism and related lipid regulatory pathways.
- **Supplementary Table 5.** Nutrient composition of the experimental formulas and sow milk.

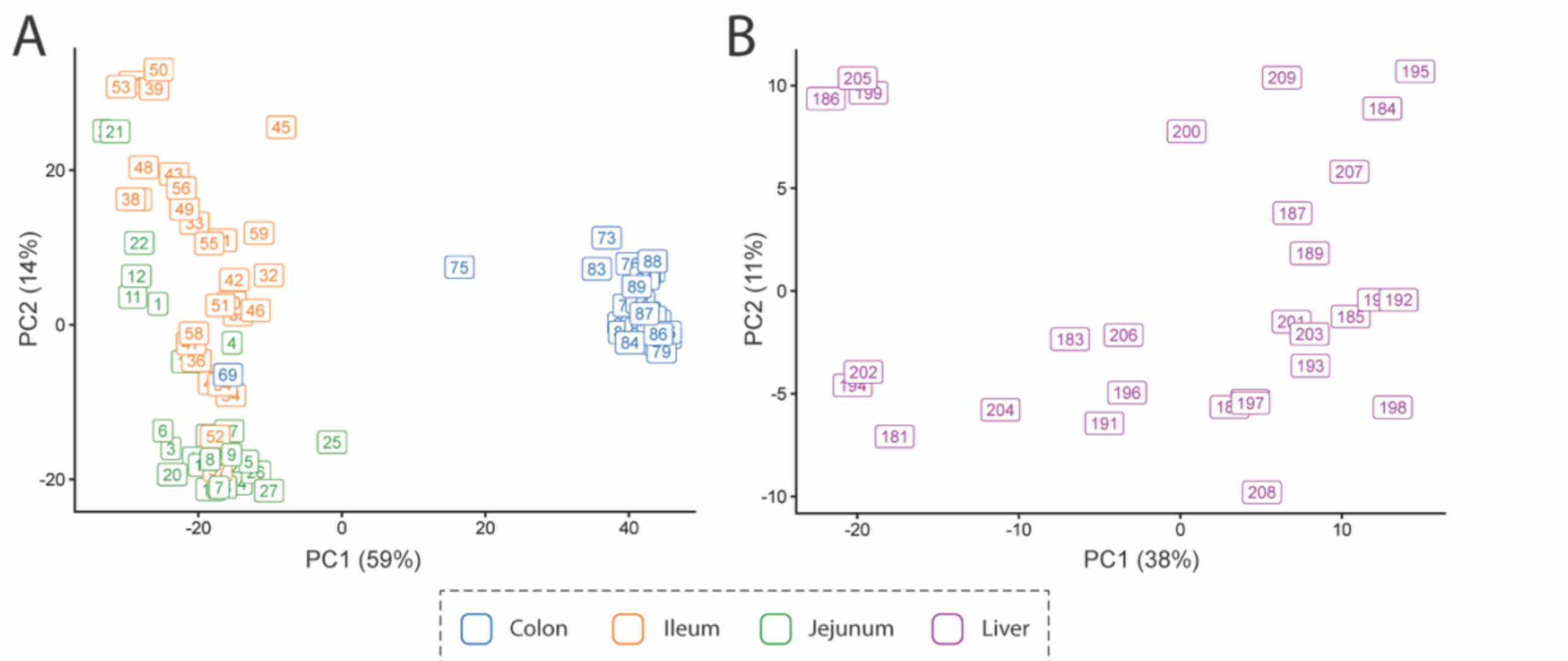

Supplementary Figure 1. Principal component analysis (PCA) of RNA-seq gene expression data used to assess sample clustering and identify potential outliers. **(A)** PCA of intestinal samples. Jejunum and ileum samples largely overlapped, indicating similar transcriptional profiles between the two regions. Two colon samples (IDs 69 and 75) were identified as outliers; accordingly, colon data for these samples are not reported in this study. No outliers were detected among jejunum or ileum samples. **(B)** PCA of liver samples showing consistent clustering with no apparent outliers. Sample ID are labeled for each point.

Supplementary Table 1. Differential expression of genes involved in cholesterol biosynthesis and regulatory pathways.

Tissue	Gene symbol	Gene name	Protein (abbr.)	BaseMean	Shrunken LFC	Adj. p-value	Higher in	Region pattern	Functional category
Liver	*ACAT2*	acetyl-CoA acetyltransferase 2 (acetoacetyl CoA thiolase)	ACAT2	106.481	1.718	4.16E-10	Formula-fed	—	Upstream of mevalonate pathway
	HMGCS1	3-hydroxy-3-methylglutaryl-CoA synthase 1	HMGCS1	586.923	2.491	3.63E-14	Formula-fed	—	Mevalonate pathway
	HMGCR	3-hydroxy-3-methylglutaryl-CoA reductase	HMGCR	258.914	2.132	5.29E-11	Formula-fed	—	Mevalonate pathway
	MVK	mevalonate kinase	MVK	678.852	1.639	2.49E-23	Formula-fed	—	Mevalonate pathway
	MVD	mevalonate diphosphate decarboxylase	MVD	39.530	1.127	4.16E-06	Formula-fed	—	Mevalonate pathway
	IDI1	isopentenyl-diphosphate delta-isomerase 1	IDI1	475.152	2.968	2.02E-13	Formula-fed	—	Mevalonate pathway
	FDPS	farnesyl diphosphate synthase	FPPS	46.983	0.920	2.80E-03	Formula-fed	—	Squalene synthesis
	FDFT1	farnesyl-diphosphate farnesyltransferase 1	SQS	455.915	0.883	6.02E-06	Formula-fed	—	Squalene synthesis
	SQLE	squalene epoxidase	SQLE	384.739	2.465	1.69E-10	Formula-fed	—	Squalene synthesis
	CYP51A1	lanosterol 14-alpha demethylase (cytochrome P450 family 51 subfamily A member 1)	CYP51	524.874	1.595	7.07E-07	Formula-fed	—	Sterol core formation
	TM7SF2	transmembrane 7 superfamily member 2	C14SR	116.412	0.707	5.37E-05	Formula-fed	—	Sterol core formation
	MSMO1	methylsterol monooxygenase 1	MSMO1	2428.798	2.117	2.97E-13	Formula-fed	—	Sterol core formation
	EBP	emopamil-binding protein	EBP	233.228	2.224	7.12E-20	Formula-fed	—	Terminal cholesterol formation
	SC5D	sterol-C5-desaturase	SC5D	229.585	1.935	3.52E-23	Formula-fed	—	Terminal cholesterol formation
	DHCR24	24-dehydrocholesterol reductase	DHCR24	1597.484	0.653	4.68E-06	Formula-fed	—	Terminal cholesterol formation
	SREBF2	sterol regulatory element binding transcription factor 2	SREBP-2	120.647	0.630	1.98E-04	Formula-fed	—	Cholesterol synthesis regulation (primary)
	INSIG1	insulin induced gene 1	INSIG-1	453.834	2.286	5.93E-48	Formula-fed	—	SREBP-2 regulation
	ERG28	ergosterol biosynthesis 28 homolog	ERG28	604.349	0.875	1.24E-06	Formula-fed	—	Cholesterol biosynthesis support
Small intestine (jejunum + ileum pooled)	*ACAT2*	acetyl-CoA acetyltransferase 2 (acetoacetyl CoA thiolase)	ACAT2	89.372	1.732	4.62E-23	Formula-fed	Both sites	Upstream of mevalonate pathway
	HMGCS1	3-hydroxy-3-methylglutaryl-CoA synthase 1	HMGCS1	553.333	1.424	9.31E-16	Formula-fed	Both sites	Mevalonate pathway
	HMGCR	3-hydroxy-3-methylglutaryl-CoA reductase	HMGCR	205.714	0.817	2.19E-08	Formula-fed	Both sites	Mevalonate pathway
	MVK	mevalonate kinase	MVK	85.577	1.014	1.49E-11	Formula-fed	Both sites	Mevalonate pathway
	MVD	mevalonate diphosphate decarboxylase	MVD	36.196	0.825	2.65E-07	Formula-fed	Both sites	Mevalonate pathway
	IDI1	isopentenyl-diphosphate delta-isomerase 1	IDI1	141.317	1.515	5.83E-15	Formula-fed	Both sites	Mevalonate pathway
	FDFT1	farnesyl-diphosphate farnesyltransferase 1	SQS	229.285	1.243	1.30E-14	Formula-fed	Both sites	Squalene synthesis
	SQLE	squalene epoxidase	SQLE	233.129	1.676	1.45E-16	Formula-fed	Both sites	Squalene synthesis
	MSMO1	methylsterol monooxygenase 1	MSMO1	665.795	1.004	2.02E-06	Formula-fed	Ileum-dominant	Sterol core formation
	HSD17B7	hydroxysteroid 17-beta dehydrogenase 7	HSD17B7	118.635	1.239	4.02E-14	Formula-fed	Both sites	Sterol core formation
	EBP	emopamil-binding protein	EBP	32.858	1.412	1.29E-13	Formula-fed	Both sites	Terminal cholesterol formation
	DHCR7	7-dehydrocholesterol reductase	DHCR7	10.590	0.855	2.22E-04	Formula-fed	Ileum-dominant	Terminal cholesterol formation
	SREBF1	sterol regulatory element binding transcription factor 1	SREBP-1	35.913	-1.870	1.43E-14	Sow-fed	Both sites	Global lipid synthesis regulation
	INSIG2	insulin induced gene 2	INSIG-2	62.413	-1.021	1.71E-11	Sow-fed	Jejunum-dominant	SREBP-2 regulation
	SMARCD3	SWI/SNF related BAF chromatin remodeling complex subunit D3	BAF60C	1.608	-0.611	3.72E-02	Sow-fed	Shared across site	SREBP-2 regulation
	ARV1	ARV1 fatty acid homeostasis modulator	ARV1	44.106	-0.724	3.40E-11	Sow-fed	Jejunum-dominant	SREBP-2 regulation
	PRKAA2	protein kinase AMP-activated catalytic subunit alpha 2	AMPKα	13.584	-0.934	2.75E-05	Sow-fed	Ileum-dominant	SREBP-2 regulation
	HNF1A	HNF1 homeobox A	HNF-1α	13.284	-0.640	1.13E-04	Sow-fed	Ileum-dominant	SREBP-2 regulation
Jejunum	*ACAT2*	acetyl-CoA acetyltransferase 2 (acetoacetyl CoA thiolase)	ACAT2	93.228	1.549	1.70E-17	Formula-fed	—	Upstream of mevalonate pathway
	HMGCS1	3-hydroxy-3-methylglutaryl-CoA synthase 1	HMGCS1	587.161	1.028	8.78E-08	Formula-fed	—	Mevalonate pathway
	HMGCR	3-hydroxy-3-methylglutaryl-CoA reductase	HMGCR	203.289	0.590	1.68E-03	Formula-fed	—	Mevalonate pathway
	MVK	mevalonate kinase	MVK	80.899	1.279	1.88E-12	Formula-fed	—	Mevalonate pathway
	MVD	mevalonate diphosphate decarboxylase	MVD	37.605	0.872	3.29E-05	Formula-fed	—	Mevalonate pathway
	IDI1	isopentenyl-diphosphate Delta-isomerase 1 (IPP isomerase 1)	IDI1	142.590	1.031	1.23E-05	Formula-fed	—	Mevalonate pathway
	FDFT1	farnesyl-diphosphate farnesyltransferase 1	SQS	223.135	1.013	1.57E-06	Formula-fed	—	Squalene synthesis
	SQLE	squalene epoxidase	SQLE	205.090	1.214	3.56E-10	Formula-fed	—	Squalene synthesis
	HSD17B7	hydroxysteroid 17-beta dehydrogenase 7 (3-keto-steroid reductase)	HSD17B7	138.233	1.119	3.85E-07	Formula-fed	—	Sterol core formation
	LBR	lamin B receptor	LBR	105.657	0.704	4.94E-07	Formula-fed	—	Terminal cholesterol formation
	EBP	emopamil-binding protein	EBP	33.351	1.032	5.64E-07	Formula-fed	—	Terminal cholesterol formation
	DHCR24	24-dehydrocholesterol reductase	DHCR24	455.221	0.794	1.78E-07	Formula-fed	—	Terminal cholesterol formation
	SREBF1	sterol regulatory element binding transcription factor 1	SREBP-1	35.098	-2.070	2.58E-13	Sow-fed	—	Global lipid synthesis regulation
	INSIG1	insulin induced gene 1	INSIG-1	220.226	-0.711	7.96E-03	Sow-fed	—	SREBP-2 regulation
	INSIG2	insulin induced gene 2	INSIG-2	66.150	-1.507	1.78E-20	Sow-fed	—	SREBP-2 regulation
	ARV1	ARV1 fatty acid homeostasis modulator	ARV1	43.216	-0.767	1.04E-07	Sow-fed	—	SREBP-2 regulation
	SEC23A	SEC23 homolog A, COPII coat complex component	SEC23A	347.997	-0.765	7.70E-06	Sow-fed	—	SREBP-2 regulation
	SEC24D	SEC24 homolog D, COPII coat complex component	SEC24D	321.676	-0.695	2.39E-05	Sow-fed	—	SREBP-2 regulation
Ileum	*ACAT2*	acetyl-CoA acetyltransferase 2 (acetoacetyl CoA thiolase)	ACAT2	85.273	1.828	4.02E-09	Formula-fed	—	Upstream of mevalonate pathway
	HMGCS1	3-hydroxy-3-methylglutaryl-CoA synthase 1	HMGCS1	517.768	1.852	3.74E-11	Formula-fed	—	Mevalonate pathway
	HMGCR	3-hydroxy-3-methylglutaryl-CoA reductase	HMGCR	207.186	1.009	3.80E-06	Formula-fed	—	Mevalonate pathway
	MVK	mevalonate kinase	MVK	89.856	0.657	3.22E-03	Formula-fed	—	Mevalonate pathway

Tissue	Gene symbol	Gene name	Protein (abbr.)	BaseMean	Shrunken LFC	Adj. p-value	Higher in	Region pattern	Functional category
	MVD	mevalonate diphosphate decarboxylase	MVD	34.649	0.649	5.39E-03	Formula-fed	—	Mevalonate pathway
	IDI1	isopentenyl-diphosphate delta-isomerase 1 (IPP isomerase 1)	IDI1	139.527	1.998	1.65E-12	Formula-fed	—	Mevalonate pathway
	FDPS	farnesyl diphosphate synthase	FPPS	31.636	0.789	1.24E-03	Formula-fed	—	Squalene synthesis
	FDFT1	farnesyl-diphosphate farnesyltransferase 1	SQS	234.342	1.423	2.36E-09	Formula-fed	—	Squalene synthesis
	SQLE	squalene epoxidase	SQLE	259.867	2.192	1.24E-10	Formula-fed	—	Squalene synthesis
	CYP51A1	lanosterol 14-alpha demethylase (cytochrome P450 family 51 subfamily A member 1)	CYP51	388.936	1.382	8.96E-10	Formula-fed	—	Sterol core formation
	MSMO1	methylsterol monooxygenase 1	MSMO1	680.205	1.945	1.40E-11	Formula-fed	—	Sterol core formation
	HSD17B7	hydroxysteroid 17-beta dehydrogenase 7 (3-keto-steroid reductase)	HSD17B7	98.915	1.284	3.16E-08	Formula-fed	—	Sterol core formation
	EBP	emopamil-binding protein	EBP	32.262	1.819	4.81E-08	Formula-fed	—	Terminal cholesterol formation
	SC5D	sterol-C5-desaturase	SC5D	51.743	1.294	1.27E-06	Formula-fed	—	Terminal cholesterol formation
	DHCR7	7-dehydrocholesterol reductase	DHCR7	8.773	1.288	7.27E-04	Formula-fed	—	Terminal cholesterol formation
	SREBF1	sterol regulatory element binding transcription factor 1	SREBP-1	36.470	-1.402	1.33E-06	Sow-fed	—	Global lipid synthesis regulation
	SREBF2	sterol regulatory element binding transcription factor 2	SREBP-2	134.224	0.649	2.62E-03	Formula-fed	—	Cholesterol synthesis regulation (primary)
	INSIG1	insulin induced gene 1	INSIG-1	267.129	1.430	1.03E-06	Formula-fed	—	SREBP-2 regulation
	PRKAA2	protein kinase AMP-activated catalytic subunit alpha 2	AMPKα	16.338	-1.287	2.74E-05	Sow-fed	—	SREBP-2 regulation
	HNF1A	HNF1 homeobox A	HNF-1α	13.543	-0.829	4.32E-04	Sow-fed	—	SREBP-2 regulation

Differential expression was defined as an absolute fold change >1.5 with an adjusted P value <0.05. Log_2 fold changes were estimated using adaptive shrinkage and are reported as 'shrunken LFC'. For small-intestine data, the pooled analysis represents a contrast corresponding to the equally weighted mean of the two formula groups relative to the sow-fed group. Among comparisons showing a significant pooled formula-fed versus sow-fed difference, the corresponding contrast between the two isocaloric formula-fed groups differing in whey protein source was not significant. For genes differentially expressed in the pooled small-intestine analysis, the 'regional pattern' column summarizes site-specific results: Both sites, significant in both jejunum and ileum; Ileum-dominant, significant only in ileum; Jejunum-dominant, significant only in jejunum; Shared across sites, significant in the pooled analysis but not individually significant in either site. This field applies only to pooled small-intestine analyses.

Supplementary Table 2. Differential expression of genes involved in cholesterol transport and associated lipoprotein assembly.

Tissue	Gene symbol	Gene name	Protein (abbr.)	BaseMean	Shrunken LFC	Adj. p-value	Higher in	Region pattern	Functional category
Liver	*NPC1L1*	NPC1 like intracellular cholesterol transporter 1	NPC1L1	19.078	2.082	1.97E-05	Formula-fed	—	Cholesterol transporter (uptake, bile → hepatocyte)
	ABCG5	ATP binding cassette subfamily G member 5	ABCG5	147.289	-0.703	2.20E-04	Sow-fed	—	Cholesterol transporter (efflux, hepatocyte → bile)
	LDLR	low density lipoprotein receptor	LDL-R	652.865	0.764	5.42E-04	Formula-fed	—	LDL and chylomicron remnants uptake receptor (blood → hepatocyte)
	CIDEB	cell death inducing DFFA like effector B	CIDEB	465.502	0.766	9.92E-07	Formula-fed	—	VLDL secretion/lipid loading
	APOE	apolipoprotein E	ApoE	1561.122	0.867	2.95E-13	Formula-fed	—	Lipoprotein metabolism (Remnant clearance)
	APOA4	apolipoprotein A4	ApoA-IV	188.557	2.539	4.30E-05	Formula-fed	—	Lipoprotein assembly and export (mainly produced in intestine)
Small intestine (jejunum+ ileum pooled)	*SCARB1*	scavenger receptor class B member 1	SR-BI	39.496	-1.289	1.12E-15	Sow-fed	Both sites	[Apical] cholesterol transporter (uptake, lumen → enterocyte); [Basolateral] HDL-mediated uptake
	SORT1	sortilin 1	Sortilin	141.755	-0.913	3.90E-13	Sow-fed	Both sites	Cholesterol uptake regulation (NPC1L1-associated trafficking)
	ABCG5	ATP binding cassette subfamily G member 5	ABCG5	71.107	-1.884	4.24E-16	Sow-fed	Both sites	[Apical] sterol/cholesterol efflux transporter (enterocyte → lumen)
	ABCG8	ATP binding cassette subfamily G member 8	ABCG8	43.959	-0.805	2.56E-05	Sow-fed	Jejunum-dominant	[Apical] sterol/cholesterol efflux transporter (enterocyte → lumen)
	NPC2	NPC intracellular cholesterol transporter 2	NPC2	159.991	-1.411	2.14E-09	Sow-fed	Both sites	Cholesterol trafficking (intracellular cholesterol transport)
	LIPA	lipase A, lysosomal acid type	LAL	489.427	-1.800	2.46E-14	Sow-fed	Both sites	Cholesterol trafficking (lysosomal cholesterol ester hydrolysis)
	APOA4	apolipoprotein A4	ApoA-IV	2293.936	-0.593	1.50E-02	Sow-fed	Jejunum-dominant	Chylomicron-associated lipoprotein assembly
	SDR42E2	short chain dehydrogenase/reductase family 42E, member 2	SDR42E2	1.201	-0.741	2.15E-02	Sow-fed	Shared across site	Chylomicron assembly and export
	APOB	apolipoprotein B	ApoB	2722.788	-2.137	9.12E-21	Sow-fed	Both sites	Chylomicron-associated lipoprotein assembly
	APOA1	apolipoprotein A1	ApoA-I	12178.283	-2.613	1.11E-12	Sow-fed	Both sites	HDL assembly and cholesterol efflux; chylomicron-associated lipoprotein assembly
	ABCA1	ATP binding cassette subfamily A member 1	ABCA1	134.845	-2.129	6.03E-22	Sow-fed	Both sites	HDL assembly and cholesterol efflux
	LCAT	lecithin-cholesterol acyltransferase	LCAT	7.545	0.721	2.44E-02	Formula-fed	Shared across site	HDL maturation and cholesterol esterification (mainly produced in liver)
	LDLR	low density lipoprotein receptor	LDLR	672.432	1.611	5.98E-19	Formula-fed	Both sites	[Basolateral] LDL uptake receptor (blood → enterocyte)
	PCSK9	proprotein convertase subtilisin/kexin type 9	PCSK9	2.977	1.315	9.85E-04	Formula-fed	Jejunum-dominant	Lipoprotein uptake regulation (promote LDLR degradation)
	MYLIP	myosin regulatory light chain interacting protein	IDOL	25.640	-1.959	5.06E-18	Sow-fed	Both sites	Lipoprotein uptake regulation (promote LDLR degradation)
	LRPAP1	LDL receptor related protein associated protein 1	RAP	30.166	-0.985	2.54E-07	Sow-fed	Both sites	Lipoprotein uptake regulation (LDLR-family receptor chaperone)
	APOC2	apolipoprotein C2	ApoC-II	418.335	-4.603	8.42E-10	Sow-fed	Ileum-dominant	Lipoprotein metabolism (lipoprotein lipase activation)
	APOE	apolipoprotein E	ApoE	24.181	-0.866	6.27E-03	Sow-fed	Ileum-dominant	Lipoprotein metabolism: remnant clearance (mainly produced in liver)
Jejunum	*SCARB1*	scavenger receptor class B member 1	SR-BI	35.571	-1.490	6.52E-14	Sow-fed	—	[Apical] cholesterol transporter (uptake, lumen → enterocyte); [Basolateral] HDL-mediated uptake
	SORT1	sortilin 1	Sortilin	134.658	-0.760	2.83E-12	Sow-fed	—	Cholesterol uptake regulation (NPC1L1-associated trafficking)
	ABCG5	ATP binding cassette subfamily G member 5	ABCG5	83.264	-2.489	1.58E-22	Sow-fed	—	[Apical] sterol/cholesterol efflux transporter (enterocyte → lumen)
	ABCG8	ATP binding cassette subfamily G member 8	ABCG8	47.697	-1.111	6.16E-07	Sow-fed	—	[Apical] sterol/cholesterol efflux transporter (enterocyte → lumen)
	NPC2	NPC intracellular cholesterol transporter 2	NPC2	92.959	-1.187	1.42E-06	Sow-fed	—	Cholesterol trafficking (intracellular cholesterol transport)
	LIPA	lipase A, lysosomal acid type	LAL	348.965	-1.887	3.46E-15	Sow-fed	—	Cholesterol trafficking (lysosomal cholesterol ester hydrolysis)
	APOA4	apolipoprotein A4	ApoA-IV	2441.304	-1.364	3.52E-05	Sow-fed	—	Chylomicron-associated lipoprotein assembly
	APOB	apolipoprotein B	ApoB	2594.586	-2.566	1.95E-21	Sow-fed	—	Chylomicron-associated lipoprotein assembly
	CIDEB	cell death inducing DFFA like effector B	CIDEB	274.153	-0.952	9.85E-05	Sow-fed	—	Chylomicron maturation/lipid loading
	MTTP	microsomal triglyceride transfer protein	MTP	1976.842	-0.825	1.47E-04	Sow-fed	—	Lipoprotein metabolism: assembly
	APOA1	apolipoprotein A1	ApoA-I	13675.987	-3.513	1.13E-11	Sow-fed	—	HDL assembly and cholesterol efflux; intestinal chylomicron-associated lipoprotein assembly
	ABCA1	ATP binding cassette subfamily A member 1	ABCA1	174.706	-2.990	4.30E-52	Sow-fed	—	HDL assembly and cholesterol efflux
	LDLR	low density lipoprotein receptor	LDLR	811.209	1.376	2.02E-10	Formula-fed	—	[Basolateral] LDL uptake receptor (blood → enterocyte)
	PCSK9	proprotein convertase subtilisin/kexin type 9	PCSK9	3.608	1.110	1.13E-02	Formula-fed	—	Lipoprotein uptake regulation (promote LDLR degradation)
	MYLIP	myosin regulatory light chain interacting protein	IDOL	34.864	-2.419	1.10E-16	Sow-fed	—	Lipoprotein uptake regulation (promote LDLR degradation)
	LRPAP1	LDL receptor related protein associated protein 1	RAP	24.765	-0.662	8.13E-03	Sow-fed	—	Lipoprotein uptake regulation (LDLR-family receptor chaperone)
Ileum	SCARB1	scavenger receptor class B member 1	SR-BI	43.063	-0.925	3.28E-05	Sow-fed	—	[Apical] cholesterol transporter (uptake, lumen → enterocyte); [Basolateral] HDL-mediated uptake
	SORT1	sortilin 1	Sortilin	147.862	-0.950	3.30E-05	Sow-fed	—	Cholesterol uptake regulation (NPC1L1-associated trafficking)
	ABCG5	ATP binding cassette subfamily G member 5	ABCG5	58.538	-0.871	2.21E-03	Sow-fed	—	[Apical] sterol/cholesterol efflux transporter (enterocyte → lumen)
	NPC2	NPC intracellular cholesterol transporter 2	NPC2	224.682	-1.573	4.91E-06	Sow-fed	—	Cholesterol trafficking: intracellular cholesterol transport
	LIPA	lipase A, lysosomal acid type	LAL	623.641	-1.617	3.27E-06	Sow-fed	—	Cholesterol trafficking: lysosomal cholesterol ester hydrolysis
	APOB	apolipoprotein B	ApoB	2828.056	-1.434	5.66E-06	Sow-fed	—	Chylomicron-associated lipoprotein assembly
	APOA1	apolipoprotein A1	ApoA-I	10562.332	-1.024	1.89E-03	Sow-fed	—	HDL assembly and cholesterol efflux; intestinal chylomicron-associated lipoprotein assembly
	ABCA1	ATP binding cassette subfamily A member 1	ABCA1	94.395	-0.721	3.78E-04	Sow-fed	—	HDL assembly and cholesterol efflux
	APOA2	apolipoprotein A2	ApoA-II	2.534	0.761	3.75E-02	Formula-fed	—	HDL-associated lipoprotein metabolism
	LDLR	low density lipoprotein receptor	LDLR	533.556	1.779	4.78E-10	Formula-fed	—	[Basolateral] LDL uptake receptor (blood → enterocyte)
	MYLIP	myosin regulatory light chain interacting protein	IDOL	16.325	-1.197	1.93E-05	Sow-fed	—	Lipoprotein uptake regulation (promote LDLR degradation)
	LRPAP1	LDL receptor related protein associated protein 1	RAP	35.278	-1.123	6.36E-05	Sow-fed	—	Lipoprotein uptake regulation (LDLR-family receptor chaperone)

Tissue	Gene symbol	Gene name	Protein (abbr.)	BaseMean	Shrunken LFC	Adj. p-value	Higher in	Region pattern	Functional category
	APOC2	apolipoprotein C2	ApoC-II	779.105	-5.310	4.47E-08	Sow-fed	—	Lipoprotein metabolism (lipoprotein lipase activation)
	APOE	apolipoprotein E	ApoE	42.754	-1.146	8.16E-03	Sow-fed	—	Lipoprotein metabolism: remnant clearance (mainly produced in liver)
	SOAT1	sterol O-acyltransferase 1	ACAT1	14.242	0.637	3.69E-02	Formula-fed	—	Cholesterol esterification

Differential expression was defined as an absolute $\log_2$ fold change >1.5 with an adjusted P value <0.05. Log_2 fold changes were estimated using adaptive shrinkage and are reported as ‘shrunken LFC’. For small-intestine data, the pooled analysis represents a contrast corresponding to the equally weighted mean of the two formula groups relative to the sow-fed group. Among comparisons showing a significant pooled formula-fed versus sow-fed difference, the corresponding contrast between the two isocaloric formula-fed groups differing in whey protein source was not significant. For genes differentially expressed in the pooled small-intestine analysis, the ‘regional pattern’ column summarizes site-specific results: Both sites, significant in both jejunum and ileum; Ileum-dominant, significant only in ileum; Jejunum-dominant, significant only in jejunum; Shared across sites, significant in the pooled analysis but not individually significant in either site. This field applies only to pooled small-intestine analyses.

Supplementary Table 3. Differential expression of genes involved in bile acid biosynthesis, regulation, transport, and detoxification.

Tissue	Gene symbol	Gene name	Protein (abbr.)	BaseMean	Shrunken LFC	Adj. p-value	Higher in	Region pattern	Functional category
Liver	CYP7A1	cytochrome P450 family 7 subfamily A member 1	CYP7A1	48.025	2.564	4.36E-05	Formula-fed	—	Bile acid biosynthesis (rate-limiting)
	CYP27A1	cytochrome P450 family 27 subfamily A member 1	CYP27A1	141.140	0.605	2.51E-04	Formula-fed	—	Bile acid biosynthesis (alternative pathway)
	ABCD3	ATP binding cassette subfamily D member 3	PMP70	773.133	0.654	7.61E-03	Formula-fed	—	Bile acid biosynthesis (peroxisomal transport)
	ACOX2	acyl-CoA oxidase 2	ACOX2	176.696	0.874	1.35E-13	Formula-fed	—	Bile acid biosynthesis (peroxisomal side-chain cleavage)
	SLC51B	solute carrier family 51 subunit beta	OST-β	2.088	-2.958	4.51E-08	Sow-fed	—	Bile acid transporter (efflux, basolateral, hepatocyte → blood)
	SLCO1A2	solute carrier organic anion transporter family member 1A2	OATP1A2	72.993	1.509	3.25E-08	Formula-fed	—	Bile acid-related transporter (minor reabsorption, bile → cholangiocyte)
	NR1I2	nuclear receptor subfamily 1 group I member 2	PXR	96.621	0.706	5.19E-07	Formula-fed	—	Bile acid detoxification
	NR1I3	nuclear receptor subfamily 1 group I member 3	CAR	22.478	0.717	6.25E-04	Formula-fed	—	Bile acid detoxification
	SULT2A1	sulfotransferase family 2A member 1	SULT2A1	637.166	1.040	2.07E-08	Formula-fed	—	Bile acid detoxification
	ABCC3	ATP binding cassette subfamily C member 3	MRP3	20.650	-1.170	9.87E-06	Sow-fed	—	Bile acid efflux and detoxification
	CYP3A29	cytochrome P450 family 3 subfamily A member 29	CYP3A29	214.245	0.936	1.30E-03	Formula-fed	—	Bile acid detoxification (pig specific)
Small intestine (jejunum + ileum pooled)	CYP27A1	cytochrome P450 family 27 subfamily A member 1	CYP27A1	3.700	-3.609	5.42E-13	Sow-fed	Both sites	Bile acid biosynthesis (alternative pathway)
	ACOX2	acyl-CoA oxidase 2	ACOX2	6.974	-1.165	1.69E-10	Sow-fed	Ileum-dominant	Bile acid biosynthesis (peroxisomal side-chain cleavage)
	HSD17B4	hydroxysteroid 17-beta dehydrogenase 4	DBP	131.992	-0.838	3.51E-21	Sow-fed	Both sites	Bile acid biosynthesis (peroxisomal β-oxidation)
	NR1H4	nuclear receptor subfamily 1 group H member 4	FXR	147.768	0.877	3.32E-05	Formula-fed	Both sites	Bile-acid signaling/regulation
	FGF19	fibroblast growth factor 19	FGF19	2.488	-1.305	2.31E-03	Sow-fed	Shared across site	Bile-acid signaling/regulation
	FGFR4	fibroblast growth factor receptor 4	FGFR4	3.361	-0.956	8.95E-04	Sow-fed	Shared across site	Bile-acid signaling/regulation (FGF19 receptor; primary hepatic signaling target)
	FABP6	fatty acid binding protein 6	IBABP	637.606	2.211	4.20E-04	Formula-fed	Ileum-dominant	Bile acid trafficking
	SLC51A	solute carrier family 51 subunit alpha	OST-α	237.062	1.481	6.99E-12	Formula-fed	Both sites	Bile acid transporter (efflux, basolateral, enterocyte → blood)
	SLC51B	solute carrier family 51 subunit beta	OST-β	150.566	0.913	4.96E-08	Formula-fed	Both sites	Bile acid transporter (efflux, basolateral, enterocyte → blood)
	SLC10A2	solute carrier family 10 member 2	ASBT	31.223	1.538	1.19E-04	Formula-fed	Ileum-dominant	Bile acid transporter (uptake, apical, lumen → enterocyte)
	NR1I3	nuclear receptor subfamily 1 group I member 3	CAR	3.045	-0.947	3.28E-03	Sow-fed	Jejunum-dominant	Bile acid detoxification
	CYP3A29	cytochrome P450 family 3 subfamily A member 29	CYP3A29	135.644	-2.906	1.81E-09	Sow-fed	Both sites	Bile acid detoxification
	ABCC2	ATP binding cassette subfamily C member 2	MRP2	419.448	-0.937	1.93E-05	Sow-fed	Both sites	Bile acid efflux and detoxification
	CYP3A22	cytochrome P450 family 3 subfamily A member 22	CYP3A22	164.570	-2.242	9.26E-15	Sow-fed	Both sites	Bile acid detoxification (pig specific)
Jejunum	CYP27A1	cytochrome P450 family 27 subfamily A member 1	CYP27A1	3.755	-4.439	3.63E-13	Sow-fed	—	Bile acid biosynthesis (alternative pathway)
	HSD3B7	hydroxy-delta-5-steroid dehydrogenase, 3 beta- and steroid delta-isomerase 7	3-βHSD7	5.347	-0.965	1.96E-03	Sow-fed	—	Bile acid biosynthesis
	CYP39A1	cytochrome P450 family 39 subfamily A member 1	CYP39A1	17.455	-0.785	3.57E-04	Sow-fed	—	Bile acid metabolism
	ABCD3	ATP binding cassette subfamily D member 3	PMP70	588.552	-1.088	5.77E-12	Sow-fed	—	Bile acid biosynthesis (peroxisomal transport)
	ACOX2	acyl-CoA oxidase 2	ACOX2	7.130	-0.925	3.07E-04	Sow-fed	—	Bile acid biosynthesis (peroxisomal side-chain cleavage)
	HSD17B4	hydroxysteroid 17-beta dehydrogenase 4	DBP	153.105	-0.984	1.28E-18	Sow-fed	—	Bile acid biosynthesis (peroxisomal β-oxidation)
	NR1H4	nuclear receptor subfamily 1 group H member 4	FXR	78.954	0.888	2.32E-03	Formula-fed	—	Bile-acid signaling/regulation
	FGFR4	fibroblast growth factor receptor 4	FGFR4	2.430	-0.808	3.23E-02	Sow-fed	—	Bile-acid signaling/regulation (FGF19 receptor; primary hepatic signaling target)
	SLC51A	solute carrier family 51 subunit alpha	OST-α	181.430	0.965	8.38E-05	Formula-fed	—	Bile acid transporter (efflux, basolateral, enterocyte → blood)
	SLC51B	solute carrier family 51 subunit beta	OST-β	155.545	0.793	1.54E-05	Formula-fed	—	Bile acid transporter (efflux, basolateral, enterocyte → blood)
	NR1I3	nuclear receptor subfamily 1 group I member 3	CAR	4.516	-0.940	1.66E-02	Sow-fed	—	Bile acid detoxification
	CYP3A29	cytochrome P450 family 3 subfamily A member 29	CYP3A29	243.518	-4.341	1.79E-10	Sow-fed	—	Bile acid detoxification
	ABCC2	ATP binding cassette subfamily C member 2	MRP2	339.430	-1.283	2.06E-07	Sow-fed	—	Bile acid efflux and detoxification
	CYP3A22	cytochrome P450 family 3 subfamily A member 22	CYP3A22	179.880	-2.822	8.54E-12	Sow-fed	—	Bile acid detoxification (pig specific)
	VDR	vitamin D receptor	VDR	54.737	-0.655	1.33E-03	Sow-fed	—	Detoxification sensor (responsive to bile acid)
Ileum	CYP27A1	cytochrome P450 family 27 subfamily A member 1	CYP27A1	3.610	-2.654	3.55E-06	Sow-fed	—	Bile acid biosynthesis (alternative pathway)
	ACOX2	acyl-CoA oxidase 2	ACOX2	6.772	-1.198	2.76E-05	Sow-fed	—	Bile acid biosynthesis (peroxisomal side-chain cleavage)
	HSD17B4	hydroxysteroid 17-beta dehydrogenase 4	DBP	110.393	-0.605	3.99E-05	Sow-fed	—	Bile acid biosynthesis (peroxisomal β-oxidation)
	NR1H4	nuclear receptor subfamily 1 group H member 4	FXR	214.809	0.766	1.65E-03	Formula-fed	—	Bile-acid signaling/regulation
	FGFR4	fibroblast growth factor receptor 4	FGFR4	4.252	-0.718	2.82E-02	Sow-fed	—	Bile-acid signaling/regulation (FGF19 receptor; primary hepatic signaling target)
	FABP6	fatty acid binding protein 6	IBABP	1185.833	0.812	3.45E-02	Formula-fed	—	Bile acid trafficking
	SLC51A	solute carrier family 51 subunit alpha	OST-α	290.948	2.067	3.82E-12	Formula-fed	—	Bile acid transporter (efflux, basolateral, enterocyte → blood)
	SLC51B	solute carrier family 51 subunit beta	OST-β	145.171	0.897	8.92E-04	Formula-fed	—	Bile acid transporter (efflux, basolateral, enterocyte → blood)
	SLC10A2	solute carrier family 10 member 2	ASBT	45.355	3.526	4.24E-09	Formula-fed	—	Bile acid transporter (uptake, apical, lumen → enterocyte)
	ABCB1	ATP-binding cassette, sub-family B (MDR/TAP), member 1	MDR1	367.154	0.615	3.47E-03	Formula-fed	—	Xenobiotic transporter (efflux, minor bile-acid role, apical, enterocyte → lumen)
	UGT1A6	UDP glucuronosyltransferase 1 family, polypeptide A6	UGT1A6	395.157	0.671	1.87E-02	Formula-fed	—	Bile acid detoxification
	CYP3A29	cytochrome P450 family 3 subfamily A member 29	CYP3A29	27.313	-0.644	4.69E-02	Sow-fed	—	Bile acid detoxification
	ABCC3	ATP binding cassette subfamily C member 3	MRP3	14.518	0.742	3.88E-03	Formula-fed	—	Bile acid efflux and detoxification
	CYP3A22	cytochrome P450 family 3 subfamily A member 22	CYP3A22	148.022	-1.251	6.08E-05	Sow-fed	—	Bile acid detoxification (pig specific)

Differential expression was defined as an absolute fold change >1.5 with an adjusted P value <0.05. Log_2 fold changes were estimated using adaptive shrinkage and are reported as 'shrunken LFC'. For small-intestine data, the pooled analysis represents a contrast corresponding to the equally weighted mean of the two formula groups relative to the sow-fed group. Among comparisons showing a significant pooled formula-fed versus sow-fed difference, the corresponding contrast between the two isocaloric formula-fed groups differing in whey protein source was not significant. For genes differentially expressed in the pooled small-intestine analysis, the 'regional pattern' column summarizes site-specific results: Both sites, significant in both jejunum and ileum; Ileum-dominant, significant only in ileum; Jejunum-dominant, significant only in jejunum; Shared across sites, significant in the pooled analysis but not individually significant in either site. This field applies only to pooled small-intestine analyses.

Supplementary Table 4. Differential expression of genes involved in other lipid metabolic pathways, including fatty acid uptake, intracellular trafficking, triacylglycerol metabolism and related lipid regulatory pathways.

Tissue	Gene symbol	Gene name	Protein (abbr.)	BaseMean	Shrunken LFC	Adj. p-value	Higher in	Regional pattern
Liver	ACSL4	acyl-CoA synthetase long chain family member 4	ACSL4	259.822	-1.072	3.61E-06	Sow-fed	—
	AKR1C1	aldo-keto reductase family 1, member C1	20-α-HSD	565.315	0.663	1.20E-03	Formula-fed	—
	ANGPTL8	angiopoietin like 8	ANGPTL8	1.150	-3.133	1.25E-06	Sow-fed	—
	APOC3	apolipoprotein C3	ApoC-III	2266.223	0.613	7.77E-03	Formula-fed	—
	EEPD1	endonuclease/exonuclease/phosphatase family domain containing 1	EEPD1	11.066	1.193	7.40E-04	Formula-fed	—
	HMGCS2	3-hydroxy-3-methylglutaryl-CoA synthase 2	HMGCS2	16.881	1.902	6.96E-04	Formula-fed	—
	HSD17B2	hydroxysteroid 17-beta dehydrogenase 2	17β-HSD2	1028.581	0.982	9.10E-08	Formula-fed	—
	HSD3B1	hydroxy-delta-5-steroid dehydrogenase, 3 beta- and steroid delta-isomerase 1	3β-HSD1	14.965	0.663	1.48E-02	Formula-fed	—
	SDR42E1	short chain dehydrogenase/reductase family 42E, member 1	SDR42E1	40.809	0.751	5.26E-06	Formula-fed	—
	SLC27A2	solute carrier family 27 member 2	FATP2	242.373	1.857	2.58E-16	Formula-fed	—
	SLC27A4	solute carrier family 27 member 4	FATP4	8.408	-0.616	5.19E-03	Sow-fed	—
	OSBPL9	oxysterol binding protein like 9	ORP9	225.769	0.775	1.52E-10	Formula-fed	—
Small intestine (jejunum+ ileum pooled)	ACSL3	acyl-CoA synthetase long chain family member 3	ACSL3	2187.979	-1.746	5.79E-13	Sow-fed	Jejunum-dominant
	AGO1	argonaute RISC component 1	AGO1	294.434	-0.711	1.20E-23	Sow-fed	Both sites
	AKR1C1	aldo-keto reductase family 1, member C1	20-α-HSD	2.594	2.273	3.55E-04	Formula-fed	Jejunum-dominant
	APOC3	apolipoprotein C3	ApoC-III	213.404	-1.849	1.51E-06	Sow-fed	Jejunum-dominant
	APOC4	apolipoprotein C4	ApoC-IV	12.660	-2.625	1.40E-04	Sow-fed	Both sites
	APOD	apolipoprotein D	ApoD	7.632	0.901	1.61E-03	Formula-fed	Jejunum-dominant
	CYB5R1	cytochrome b5 reductase 1	CYB5R1	35.581	-0.939	9.27E-14	Sow-fed	Both sites
	DGAT1	diacylglycerol O-acyltransferase 1	DGAT1	115.692	-0.655	4.43E-06	Sow-fed	Jejunum-dominant
	EEPD1	endonuclease/exonuclease/phosphatase family domain containing 1	EEPD1	7.652	-0.628	2.29E-03	Sow-fed	Shared across sites
	FASN	fatty acid synthase	FASN	132.644	1.171	2.20E-15	Formula-fed	Both sites
	FDXR	ferredoxin reductase	FDXR	4.515	0.955	7.19E-04	Formula-fed	Jejunum-dominant
	HSD17B2	hydroxysteroid 17-beta dehydrogenase 2	17β-HSD2	97.548	-0.946	1.88E-03	Sow-fed	Jejunum-dominant
	LPL	lipoprotein lipase	LPL	278.605	3.314	2.27E-47	Formula-fed	Both sites
	OSBPL1A	oxysterol binding protein like 1A	ORP1	98.709	-0.852	1.67E-10	Sow-fed	Both sites
	OSBPL9	oxysterol binding protein like 9	ORP9	68.093	-0.601	8.60E-12	Sow-fed	Jejunum-dominant
	PRLR	prolactin receptor	PRLR	50.383	-0.739	2.34E-04	Sow-fed	Jejunum-dominant
	SLC27A2	solute carrier family 27 member 2	FATP2	9.736	-1.084	8.87E-05	Sow-fed	Both sites
	SLC27A4	solute carrier family 27 member 4	FATP4	305.951	-0.619	1.43E-04	Sow-fed	Jejunum-dominant
Jejunum	ACOT8	acyl-CoA thioesterase 8	ACOT8	20.927	-0.747	7.34E-05	Sow-fed	—
	ACSL3	acyl-CoA synthetase long chain family member 3	ACSL3	2122.850	-2.468	1.41E-18	Sow-fed	—
	AGO1	argonaute RISC component 1	AGO1	276.843	-0.661	1.06E-12	Sow-fed	—
	AKR1C1	aldo-keto reductase family 1, member C1	20-α-HSD	4.537	3.348	3.53E-05	Formula-fed	—
	APOC3	apolipoprotein C3	ApoC-III	215.337	-2.854	4.37E-07	Sow-fed	—
	APOC4	apolipoprotein C4	ApoC-IV	1.707	-2.037	2.02E-03	Sow-fed	—
	APOD	apolipoprotein D	ApoD	8.450	1.260	2.03E-03	Formula-fed	—
	CYB5R1	cytochrome b5 reductase 1	CYB5R1	30.333	-0.874	1.55E-08	Sow-fed	—
	DGAT1	diacylglycerol O-acyltransferase 1	DGAT1	119.384	-0.997	1.05E-08	Sow-fed	—
	ELOVL5	ELOVL fatty acid elongase 5	ELOVL5	73.426	-0.670	1.94E-04	Sow-fed	—
	FABP2	fatty acid binding protein 2	I-FABP	18977.721	-0.991	1.74E-03	Sow-fed	—
	FADS1	fatty acid desaturase 1	FADS1	19.215	-0.662	7.62E-03	Sow-fed	—
	FADS2	fatty acid desaturase 2	FADS2	5.721	-0.971	1.38E-03	Sow-fed	—
	FASN	fatty acid synthase	FASN	147.418	1.259	1.82E-13	Formula-fed	—
	FDX1	ferredoxin 1	FDX1	61.845	-0.623	2.41E-06	Sow-fed	—
	FDXR	ferredoxin reductase	FDXR	5.222	1.356	1.16E-03	Formula-fed	—
	GPAM	glycerol-3-phosphate acyltransferase, mitochondrial	GPAT1	108.691	0.720	9.55E-09	Formula-fed	—
	HSD17B11	estradiol 17-beta-dehydrogenase 11	17β-HSD11	121.425	-0.714	3.69E-04	Sow-fed	—
	HSD17B2	hydroxysteroid 17-beta dehydrogenase 2	17β-HSD2	49.231	-1.113	4.71E-03	Sow-fed	—
	LPL	lipoprotein lipase	LPL	334.136	3.941	5.13E-61	Formula-fed	—
	OSBP	oxysterol binding protein	OSBP	29.359	-0.591	4.09E-04	Sow-fed	—
	OSBPL1A	oxysterol binding protein like 1A	ORP1	102.990	-0.919	9.12E-06	Sow-fed	—
	OSBPL3	oxysterol binding protein like 3	ORP3	24.092	-0.802	4.73E-07	Sow-fed	—
	OSBPL9	oxysterol binding protein like 9	ORP9	65.155	-0.786	5.66E-11	Sow-fed	—
	PRLR	prolactin receptor	PRLR	63.749	-1.076	1.89E-05	Sow-fed	—
	SERINC1	serine incorporator 1	SERINC1	234.279	-0.598	8.50E-11	Sow-fed	—
	SLC27A2	solute carrier family 27 member 2	FATP2	11.358	-0.745	2.86E-02	Sow-fed	—
	SLC27A4	solute carrier family 27 member 4	FATP4	324.011	-0.977	1.33E-06	Sow-fed	—

Tissue	Gene symbol	Gene name	Protein (abbr.)	BaseMean	Shrunken LFC	Adj. p-value	Higher in	Regional pattern
Ileum	OSBPL1A	oxysterol binding protein like 1A	ORP1	93.796	-0.677	5.68E-05	Sow-fed	—
	AGO1	argonaute RISC component 1	AGO1	309.917	-0.707	1.40E-09	Sow-fed	—
	APOC4	apolipoprotein C4	ApoC-IV	23.296	-3.259	2.38E-04	Sow-fed	—
	CD36	CD36 molecule	CD36	68.679	0.838	2.43E-04	Formula-fed	—
	CYB5R1	cytochrome b5 reductase 1	CYB5R1	40.514	-0.904	9.49E-06	Sow-fed	—
	ELOVL6	ELOVL fatty acid elongase 6	ELOVL6	100.788	0.842	2.22E-03	Formula-fed	—
	FASN	fatty acid synthase	FASN	117.649	0.988	1.54E-05	Formula-fed	—
	LPL	lipoprotein lipase	LPL	223.460	2.669	3.84E-14	Formula-fed	—
	SLC27A2	solute carrier family 27 member 2	FATP2	8.074	-1.103	3.21E-03	Sow-fed	—
		acyl-coenzyme A thioesterase 1-like		66.228	0.763	5.68E-05	Formula-fed	—

Differential expression was defined as an absolute fold change >1.5 with an adjusted P value <0.05. Log_2 fold changes were estimated using adaptive shrinkage and are reported as 'shrunken LFC'. For small-intestine data, the pooled analysis represents a contrast corresponding to the equally weighted mean of the two formula groups relative to the sow-fed group. Among comparisons showing a significant pooled formula-fed versus sow-fed difference, the corresponding contrast between the two isocaloric formula-fed groups differing in whey protein source was not significant. For genes differentially expressed in the pooled small-intestine analysis, the 'regional pattern' column summarizes site-specific results: Both sites, significant in both jejunum and ileum; Ileum-dominant, significant only in ileum; Jejunum-dominant, significant only in jejunum; Shared across sites, significant in the pooled analysis but not individually significant in either site. This field applies only to pooled small-intestine analyses.

Supplementary Table 5. Nutrient composition of the ALAC formula, the WPI formula and sow milk.

	**ALAC formula** *formula made with α-lactalbumin enriched whey protein isolate*	**WPI formula** *formula made with standard whey protein isolate*	**Sow milk** *Quantified using pooled sow milk*	*Literature values*
Energy (kcal/L)	1068.2	1063.2		1140[1]
Carbohydrate *(g/L)*	56.55	56.55		
Lactose (g/L)	*55.00*	*55.00*	*54.5*	*54*[1]; *41.1-55.3*[2]
Corn starch (g/L)	*1.55*	*1.55*		
Protein (g/L)	44.30 ± 0.85	43.15 ± 0.78	46.19	42.2[1]; 48.3-50.4[2]; 54[3]
Whey:casein ratio	*49.5:50.5*	*50:50*		*50:50*[4]
α-Lactalbumin (g/L)	*18.33*	*1.83*		
β-Lactoglobulin (g/L)	*1.43*	*12.94*		
Casein glycomacropeptide (g/L)	*0.00*	*4.25*		
Fat (g/L)	70.00	70.00		71[1]; 73.3-78.4[2] ; 76[3]
Amino acid composition (g/L)				
Alanine	1.00 ± 0.00	1.66 ± 0.03	1.52	2.47[1]
Arginine	1.65 ± 0.04	1.68 ± 0.01	2.13	0.89[1]
Aspartate/asparagine	4.89 ± 0.08	3.62 ± 0.05	3.86	4.21[1]
Methionine	1.00 ± 0.04	1.12 ± 0.00	1.07	0.74[1]
Cysteine	1.16 ± 0.05	0.62 ± 0.01	0.68	0.65[1]
Glutamate/glutamine	7.80 ± 0.23	8.01 ± 0.07	9.21	6.83[1]
Glycine	0.89 ± 0.01	0.88	1.18	1.64[1]
Histidine	1.09 ± 0.00	0.97	1.32	0.65[1]
Isoleucine	2.24 ± 0.04	2.39 ± 0.08	1.96	1.94[1]
Leucine	4.07 ± 0.08	4.11 ± 0.08	3.79	3.71[1]
Lysine	3.89 ± 0.03	3.62 ± 0.04	3.60	3.10[1]
Phenylalanine	1.89 ± 0.06	1.59 ± 0.01	1.81	1.40[1]
Tyrosine	1.98 ± 0.05	1.64 ± 0.00	1.95	1.23[1]
Proline	3.70 ± 0.06	3.74 ± 0.04	4.71	5.33[1]
Serine	1.97 ± 0.05	2.03 ± 0.01	2.36	2.56[1]
Threonine	1.95 ± 0.01	2.35 ± 0.02	2.08	2.15[1]
Tryptophan	1.03 ± 0.05	0.89 ± 0.10	0.66	0.35[1]
Valine	2.12 ± 0.04	2.44 ± 0.03	2.30	2.39[1]
Taurine	166.00	166.00	165.9	

Values are summarized here to provide dietary context for the present pathway-focused secondary analysis. The full formula ingredient data, complete dietary composition and analytical methods were provided in the parent animal study[5,6]. Pooled sow milk composition was measured and used as a reference during formula design. Longitudinal milk composition from individual sows nursing sow-fed piglets was not measured. Cholesterol and phytosterol concentrations were not measured and are therefore not included in this table.

References

1. Odle, J., Lin, X., Jacobi, S.K., Kim, S.W., and Stahl, C.H. (2014). The Suckling Piglet as an Agrimedical Model for the Study of Pediatric Nutrition and Metabolism. *Annu Rev Anim Biosci* **2**, 419–444. https://doi.org/10.1146/annurev-animal-022513-114158.

2. Zhang, S., Chen, F., Zhang, Y., Lv, Y., Heng, J., Min, T., Li, L., and Guan, W. (2018). Recent progress of porcine milk components and mammary gland function. *J Anim Sci Biotechnol* **9**, 77. https://doi.org/10.1186/s40104-018-0291-8.

3. Hurley, W.L. (2015). Composition of sow colostrum and milk. In The gestating and lactating sow, C. Farmer, ed. (Wageningen Academic Publishers), pp. 193–230. https://doi.org/10.3920/978-90-8686-803-2_9.

4. J. Csapó, T. G. Martin, Z. S. Csapo-Kiss, and Z. Hazas (1996). Protein, fats, vitamin and mineral concentrations in porcine colostrum and milk from parturition to 60 days. *International Dairy Journal* **6**, 881–902. https://doi.org/10.1016/0958-6946(95)00072-0.

5. Shoff, S. et al. The role of alpha-lactalbumin in modulating tryptophan metabolism and serotonin synthesis. *NPJ Sci Food* **9**, 120 (2025). https://doi.org/10.1038/s41538-025-00497-6

6 He, X. et al. Functional gut variability drives metabolic and transcriptional features in neonatal piglets. *NPJ Sci Food* **9**, 207 (2025). https://doi.org/10.1038/s41538-025-00571-z
